# Using FlowCam and molecular techniques to assess the diversity of Cyanobacteria species in water used for food production

**DOI:** 10.1038/s41598-022-23818-1

**Published:** 2022-11-08

**Authors:** Mulalo I. Mutoti, Afam I. O. Jideani, Jabulani R. Gumbo

**Affiliations:** 1 Department of Earth Sciences, Faculty of Science, Engineering and Agriculture, University of Venda, Private Bag, Thohoyandou, X50500950 South Africa; 2 Department of Food Science and Technology, Faculty of Science, Engineering and Agriculture, University of Venda, Private Bag X5050, Thohoyandou, 0950 South Africa; 3 Special Interest Group Post Harvest Handling, ISEKI-Food Association, Muthgasse 18, 1190 Vienna, Austria

**Keywords:** Microbiology, Environmental sciences

## Abstract

Globally, the occurrence of cyanobacteria in water currently remains an important subject as they produce cyanotoxins that pose threat to human health. Studies on the contamination of maize meals during mill grinding processes using cyanobacteria-contaminated water have not been conducted. The present study aimed to assess the diversity of cyanobacteria in the samples (process water, uncooked maize meal, and cooked maize meal (porridge)). Polymerized Chain Reaction (PCR) and Advanced digital flow cytometry (FlowCAM) were used to detect and identify cyanobacterial species available in these samples. 16S Primers (forward and reverse) tailed with Universal Sequences were used for amplification and sequencing of full-length 16S rRNA genes from cyanobacteria found in all samples. Cyanobacterial species from order Nostocales, Pseudanabaenales, Oscillatoriales Chroococcales, Synechococcales, and unclassified cyanobacterial order, some of which have the potential to produce cyanotoxins were amplified and identified in process water, raw maize meal and porridge samples using PCR. Images of the genus *Microcystis, Phormidium*, and *Leptolyngbya* were captured in process water samples using FlowCAM. These findings show the presence of cyanobacteria species in process water used for maize meal and the absence in cooked maize meal. The presence of cyanobacteria in process water is likely another route of human exposure to cyanotoxins.

## Introduction

Cyanobacteria occur in all surface waters where physical and chemical conditions are favourable to their growth, presenting a significant public health hazard across the world^1,2^. When these conditions are favourable, cyanobacteria form blooms and increase biomass accumulation and scum^3^. As secondary metabolites, they produce and release a variety of compounds known as cyanotoxins that pose threat to human health^2,4,5^. The risk to human health comes after direct or indirect contact with water or food contaminated with these compounds^3,6–8^. However, cyanotoxins are not the only compounds that are produced by cyanobacteria, cyanobacteria also produce taste and odour compounds such as geosmin and 2-methylisoborneol (MIB)^9,10^. *Anabaena* sp., *Aphanizomenon* sp., *Lyngbya* sp., *Microcystis* sp., *Oscillatoria* sp. and *Phormidium* sp. are cyanobacterial genera that are known to produce taste and odour compounds as well as cyanotoxins^9^. Different types of toxins are produced by cyanobacteria and these include hepatotoxins also known as liver toxins (e.g., microcystins, cylindrospermopsin), neurotoxins also known as nervous system toxins (e.g., anatoxins, saxitoxins), dermatotoxins also known as skin toxins (e.g., lyngbyatoxins) and irritant toxins or toxins that affect the skin and mucous membranes (e.g., lipopolysaccharides)^11,12^. Owing to their stability and resilience to biological and chemical breakdown, their widespread occurrence and their potential to reach high concentrations in blooms and scums, microcystins (MCs) have become the most common studied toxins globally^13–15^. MCs are hepatotoxins that are commonly produced by *Microcystis* and other genera, including *Anabaena*, *Nostoc*, *Nodularia*, and *Planktothrix*^16–18^.

Various human exposure routes to cyanobacteria have been reported in previous studies^2,3,7,19,20^, and these include exposure through ingestion of contaminated drinking water which may also occur during recreational water activities such as swimming^7^. Consumption of food affected by cyanobacteria-contaminated water has also been reported as another human exposure route to cyanobacteria^8^. Another exposure is through inhalation of cyanobacteria-contaminated water during swimming or showering^20^. However, amongst these exposure routes, the interest of the current paper is in the exposure route through ingestion of cyanobacteria-contaminated water and food. People in underdeveloped areas have been reported to use untreated water from sources for drinking and other domestic uses. Such water has been reported to have pathogenic bacteria, including cyanobacteria^1^. In rural areas, 20 to 25 L containers are used to collect and store water for everyday use. Jagals et al.^1^ reported that the way water from these containers is being used by households poses health threats to the users. Cyanobacteria may develop in the container and could proliferate in container waters and eventually attach to containers’ inner sidewalls, forming biofilm, further sustaining their occurrence and proliferation.

Recommended drinking water guidelines for cyanotoxins have been established and reported in previous studies. Mutoti et al.^3^ reported recommended guideline values of 1 μg/L, 3.7 μg/L, 3 μg/L, and 3 μg/L for microcystins, anatoxin-a, cylindrospermopsin, and saxitoxins, respectively. Many studies have reported human health-related problems caused by exposure to cyanotoxins^4,7,20,21^. Most reported health problems have been linked with chronic exposure to low concentrations of microcystin when consuming water or food contaminated by microcystin as well as dermal exposure and inhalation^15,22,23^. However, chronic exposure has caused serious poisoning in humans, even leading to deaths^7^. Many cases have been reported of human poisoning by cyanotoxins in water^24–26^. An episode has been reported in Brazil, where 2000 cases of gastroenteritis and 88 deaths were reported within 42 days due to poisoning by blooms from *Anabaena* and *Microcystis* freshwater genera^27^. Chronic exposure to low quantities of cyanotoxins can lead to tumour promotion and the development of cancer in humans^20^. Mortalities have been caused by cancer in developed and developing countries worldwide^4^. In many countries, colorectal cancer is another tumour that is strongly associated with the consumption of water contaminated by microcystin. A study in China has reported that a dosage of 50 pg/ml (0.05 μg/L) of microcystins in water has multiplied the risk of colorectal cancer by 7.9%^4^.

Authorities that deal with drinking water are often faced with the challenge of treating cyanobacteria-contaminated water and the lack of skilled personnel to develop monitoring programmes and protocols for cyanobacteria toxins has been highlighted as the main challenge faced by the drinking water treatment works (DWTW)^9^. However, a guideline document (South African Water Quality Guidelines) has been developed for drinking water suppliers in South Africa to assist producers of portable water to deal with cyanobacteria and their toxins in water sources. A better understanding of treatment requirements can be achieved through monitoring of cell counts or biovolume estimates in source waters when integrated with toxin information. Advanced digital flow cytometry (FlowCAM) and Polymerized Chain Reaction PCR, amongst other methods, have been widely used to detect, identify and classify cyanobacterial species in drinking water^10,28–30^. Digital flow cytometry FlowCAM (Fluid Imaging Technologies, Scarborough, Maine U.S.A) has been used for cell counting of cyanoHABs since being used by Sieracki et al.^31^. The technology has been dominantly applied in marine and estuarine studies^32,33^ and applied less in freshwater studies^10,34^. FlowCam identifies cyanobacteria based on the shape and morphological parameters (length, area, width, area-based diameter, and equivalent spherical diameter) of their images obtained from the FlowCam equipped with a microscope and a digital camera^35^. Therefore, this tool has been used as an assessment approach to quantify the risk of exposure to cyanobacteria and their toxins that pose threats to human health. Furthermore, the classification of cyanobacteria has been carried out by previous studies using PCR^36–38^, with much effort being directed towards the assessment of cyanobacterial diversity. Cyanobacteria have been identified up to the genus level using the 16S rRNA gene sequencing^37^. However, factors such as sequencing method, amplicon targeted (locus and region), bioinformatics pipeline, thermal cycling conditions used, and choice of primers have been identified to affect the success and resultant quality of PCR-based sequencing^38^. Therefore, 16S rRNA coupled with available sequencing information gives higher detectability and allows taxonomic classification of cyanobacteria, potentially to species level^38–40^.

The current study aimed to evaluate the occurrence of cyanobacterial species in containers of water and maize meal powder and assess the health risks that these cyanobacteria can pose to households consuming maize meal. The use of cyanobacteria-contaminated water might contaminate the mill during grinding processes and pose threat to humans. The current study recognized the necessity of this assessment as cyanobacteria can comprise a mix of species, each of which may or may not produce toxins. For health risk assessment, it is fundamental to determine whether the species present in water from containers are not at levels that can pose a human health problem.

## Materials and methods

### The study area

**Figure 1:**
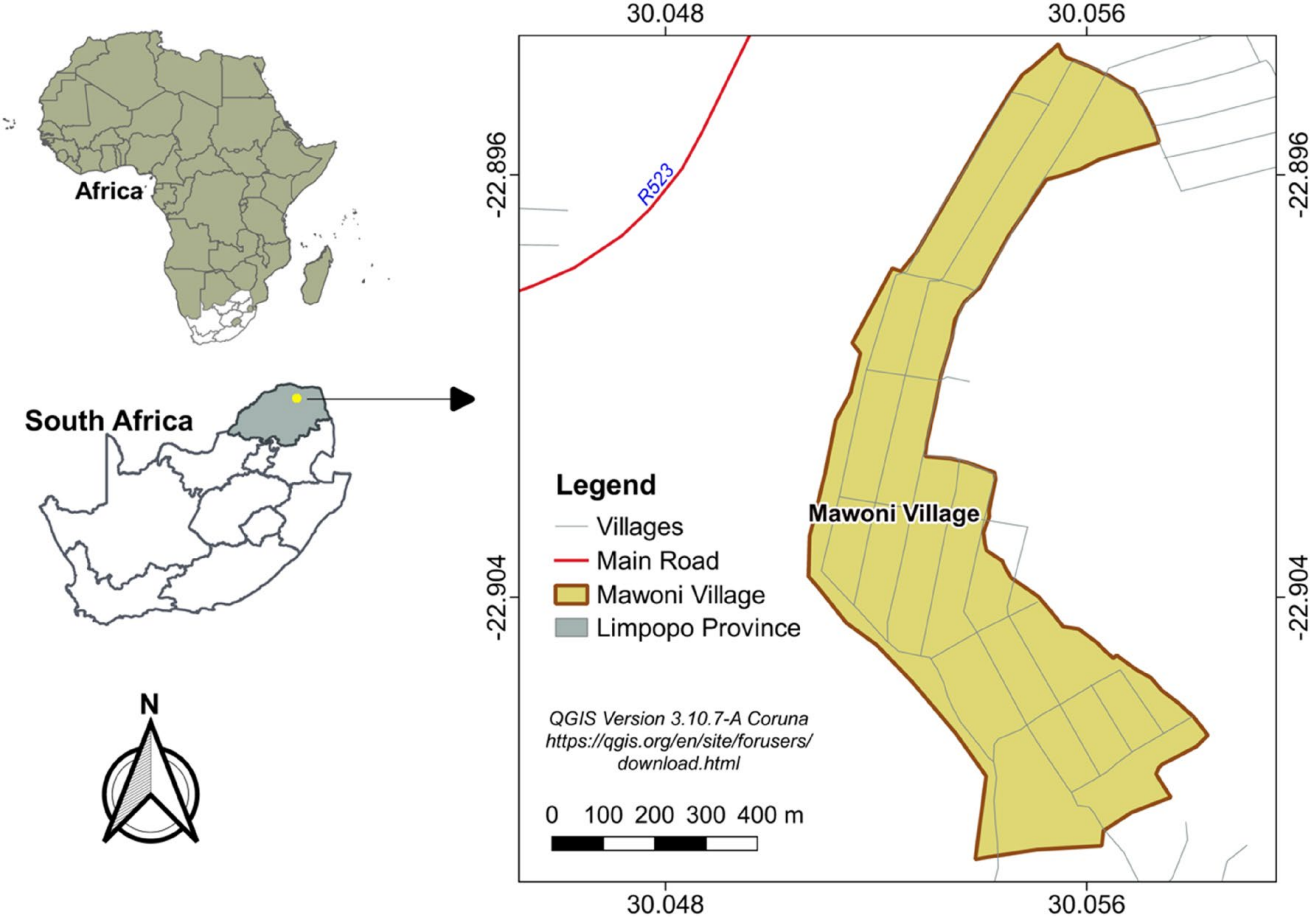
Map of the study area.

This study was conducted in Mawoni Village in Makhado Municipality, which falls under the Vhembe District in Limpopo province (Figure 1). The village is supplied with treated water from Mutshedzi dam, which the present study suspects the occurrence of cyanobacteria in such treated water. The sampling site is a mill grinding station that people from Mawoni and other surrounding villages depend on for their mill grinding. At this site, they use both cyanobacteria-contaminated water from the containers and the borehole water to soften the grains before grinding.

Live cyanobacterial samples were collected in the summer (December 2020) using Latex Free, Polypropylene Medline Basic Specimen containers with screw lids (manufactured by Medline, US). Four water samples (Table 1) were collected from two different containers. Two water samples (SM) were collected before dislodging as a free volume of water from each water container. The other two water samples (SD) were collected after the biofilm on the sidewalls of the container was dislodged. This was done by brushing the sidewalls of the container followed by vigorous shaking. Water samples were collected from the mill grinding station that was randomly selected for the present study. The water samples were collected from containers (Figure S1) used to collect and store water. Analysis of physicochemical parameters (pH, EC, DO, Temp, Salinity and TDS) was performed in triplicate at the site during sample collection using the Accsen PC 70 Multimeter, manufactured by Accsen Instrumental in Italy. Water turbidity was measured in triplicate using the Turbicheck WL portable Turbidity meter (TB 250 WL, manufactured by Lovibond Water Testing, Germany). An infrared thermal imaging camera (LC TECH DT-8530 Infrared Thermometer, supplied by JALC Instruments, Philippines, Asia) was used to measure the temperature during the grinding mill process. Two samples of raw maize meal powder produced from the grinding processes were also collected for cyanotoxin analysis. The first sample (P1) was collected just after the cyanobacteria-contaminated water was introduced to the maize grain and the second sample (P2) was collected after the whole grinding mill process is complete. A porridge sample (P3) was also prepared for the present study using raw maize meal sample (P2). For the preparation of the porridge sample, water was boiled in a cooking pan and a small porridge was cooked. After cooling, the porridge sample was then collected. Cyanobacterial samples were then taken to the laboratory for further analysis.

**Table 1:** Names of water samples collected for cyanobacterial analysis.

Container	Sample name	
Container A	Sample before dislodging (SM-1)	GFF 1
	Sample after dislodging (SD-1)	
Container B	Sample before dislodging (SM-2)	GFF 2
	Sample after dislodging (SD-2)	

### Instrumentation

Thousands of captured images can be generated from a sample taken during cyanobacterial bloom using a FlowCAM analysis^10^. The bench-top FlowCAM (8100-C, Fluid Imaging Technologies Inc., Scarborough., ME, USA) equipped with the *VisualSpreadsheet* Version 3.2.2 software and auto-focused for X10 objective was used in the present study. The taxonomic reference used in this study is van Vuuren et al.^41^. For this analysis, the FC100-µm deep flow cell (from Fluid Imaging Technologies Inc., Scarborough., ME, USA) was coupled with the X10 objective, therefore samples that consist of cells less than 100-µm diameter could pass through the flow cell without clogging. To avoid time-consuming sample processing, samples were not diluted during this analysis. Disposable 3 mL Pasteur Pipettes (supplied by Lasec Group, South Africa) were used to transfer the samples to the FlowCAM. This type of pipette helps to transfer the precise volume that equals the specific volume setting to avoid negative consequences of sedimentation and clogging. The FlowCAM was set to a flow rate of 0.2 mL/min and an auto image capture rate of 20 frames/sec. Other software settings are shown in Table S1. Before each sample was analyzed, the prime system settings were used to flush the flow assembles within the assembly. This was performed three times before each sample analysis followed by filling the assembly with deionized water to remove all the bubbles formed within the assembly during flushing. Then, the machine prime was used to draw each sample into the flow cell. These images appear as a homogenous distribution plot, plotted along the capture *x*- and capture *y*-axes in the *VisualSpreadsheet* detection window. A transect was then drawn across any portion of the distribution, which then provided a precise and unbiased subset of images for selection and identification. During this time, a random transect was selected by clicking and dragging the mouse across a portion of an entire detection window that contained plus-minus 10 000 cyanobacterial cells. Captured images were then organized in a *VisualSpreadsheet* using the image parameter sorting function. Finally, the particle length trait was used to group the images into major cyanobacteria morphotypes, which were then identified at the species level. After this, images were stored in a library.

### FlowCAM analysis

As adopted from Graham et al.^10^, Park et al.^35^, and Paulton^33^, images of cyanobacterial species were generated using the FlowCAM. The FlowCAM detects and captures cyanobacteria images in a device that has a filter tube diameter coupled with a specific magnification that counts cells, for example, a 300 µm tube filter coupled with X400 magnification or 600 µm tube filter coupled with X200 magnification^10,35^. Images are captured as the water sample flows past the filter tube inside the FlowCAM device. A 100 µm tube filter coupled with x10 magnification was used in the present study to capture images. This tube was selected because it covers the most dominant sizes of colonies that are found in water^35^. Therefore, colonies that are larger than 100 µm were excluded by filtering the sample to prevent clogging of particles in the filter tube^42^. A minimum of 2000 photo images were captured in each water sample from the sampling site.

### Polymerized chain reaction analysis

#### Isolation and cultivation

The isolation and cultivation of cyanobacteria were laboratory prepared using a modified BG11 medium as per Gumbo et al.^43^, Moza and Postolache^29^, and Valadez-Cano et al.^30^. Cyanobacteria were grown in a liquid medium (BG11). The modified BG11 medium was sterilized in 200 mL and transferred to sterile 250 mL laboratory jars under sterile conditions. Additionally, 10 mL of raw sample was added to a 250 mL laboratory jar containing the modified BG11. The laboratory jars containing cultures were incubated for 30 days under continuous light (1100 lux) fluorescent lamps at room temperature. After 30 days of incubation, the harvested cyanobacteria cells were used for identification and molecular characterization.

#### Genomic DNA extraction and PCR amplification of 16S rRNA gene

**Figure 2:**
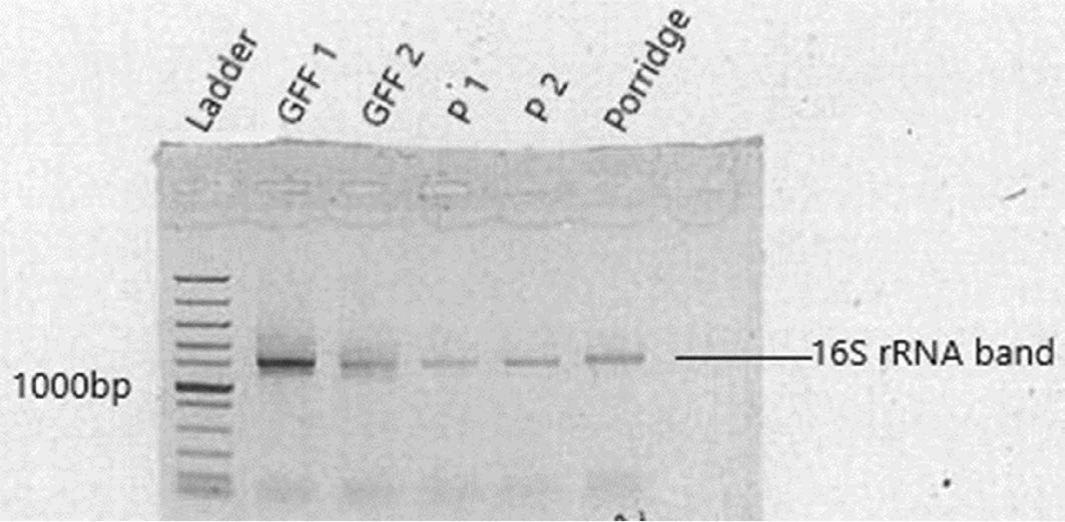
Agarose gel electrophoresis showing amplified 16S rRNA of the bacterial isolates. Lane ladder represents the 100 bp molecular ladder. The lanes (GFF1, GFF2, P1, P2 and Porridge) express the level of migration of genes on the agarose gel.

Total gDNA extractions were carried out for cultured samples, raw maize meal and porridge samples using the ZR-DuetTM DNA/RNA Miniprep DNA extraction kit supplied by Inqaba Biotech Laboratories South Africa (Pretoria, Gauteng Province). Standard techniques for sample preparation and gDNA extraction were carried out according to the instructions supplied by the manufacturer. Extracts were then stored at −20 °C until further analysis. The present study carried out the multiplexing 16S amplicons using the Barcoded Universal Primer approach. This workflow uses two rounds of PCR. The first was with universal primer-tailed 16S primers and the second PCR with PacBio Barcoded Universal Primers. With the first round of PCR, a bacterial sequencing library that targets the bacterial 16S rRNA genes was arranged using primer sets from PacBio 16S protocol (V1–V9 regions) shown in Table 2. These primers were modified by adding a 5’ block and a tail representing the PacBio universal sequences. Reactions were carried out in a 25 µL reaction containing PCR-grade water (as required for 25 µL reaction), 12.5 µL of 2X KAPA HiFi HotStart ReadyMix, 0.75 µL of each of 10 µL reverse and forward primers and 25 pg–2.5 ng of template DNA. The cycling conditions were 3 min at 95 °C, then 23 cycles of 30 sec at 95 °C, 30 s at 57 °C, 60 s at 72 °C and then followed by repeating steps 2 to 4 for a total of 20 cycles. A second-round amplification was then achieved using the Barcoded Universal F/R Primers Plate-96 available from PacBio (Inqaba Biotech Laboratories South Africa). Reactions were carried out in a 25 µL reaction containing PCR-grade water (as required for 25 µL reaction), 12.5 µL of 2X KAPA HiFi HotStart ReadyMix, 3.75 µL of 2 µM Barcoded Universal primers and 1 – 2 ng of first round PCR product. The cycling conditions were 30 sec at 95 °C, 30 sec at 57 °C, 60 sec at 72 °C and then steps 1 to 3 were repeated for a total of 20 cycles. The PCR-amplified products on agarose gel showed prominent DNA bands with approximate sizes of ≥1100 base pairs (Figure 2). All the reagents used in the present study were supplied by Inqaba Biotech Laboratories South Africa (Pretoria, Gauteng Province).

**Table 2:** Primers for cyanobacterial 16S rRNA gene amplification and sequencing.

ID	Sequence
27F27F	/5AmMC6/ gcagtcgaacatgtagctgactcaggtcac AGRGTTYGATYMTGGCTCAG
14292R	/5AmMC6/ tggatcacttgtgcaagcatcacatcgtag RGYTACCTTGTTACGACTT

*Lowercase = Universal sequence for the library construction, *Uppercase = Designed sequence for 16S rRNA.

#### Sequencing

Samples were sequenced on the Sequel system by PacBio (www.pacb.com). Raw subreads were processed through the SMRTlink (v9.0) Circular Consensus Sequences (CCS) algorithm to produce highly accurate reads (>QV40). These highly accurate reads were then processed through VSearch (https://github.com/torognes/vsearch) and taxonomic information was determined based on QIMME2. The sequencing was carried out as shown in Figure S2.

#### Phylogenetic sequence analysis

Sequences obtained in the present study were aligned using BioEdit software program for DNA sequencing (available at www.mbio.ncsu.edu.RnaseP/info/programs/BIOEDIT/bioedit.html) and Clustal W tool for sequence alignment (Version 4.0 of MEGA ANALYSIS software). Sequences were compared to the compilation of 16S rRNA gene sequences available in databases using the NCIB/BLAST^44^ (http://www.ncbi.nlm.nih.gov/BLAST/), to validate the cyanobacterial origin of sequenced samples. Sequences were then deposited in GenBank at the NCIB database. Cyanobacterial 16S rRNA gene sequences of cyanobacteria obtained in the present study and reference sequences of cyanobacteria obtained from GenBank at NCIB were used to construct the phylogenetic tree for the present study to clarify the cyanobacterial taxonomic positions of cyanobacteria belonging to the orders and unclassified cyanobacterial isolates obtained in the present study. Evolutionary distances and phylogenetic trees were calculated and constructed using the p-distance method^45^ and the neighbour-joining method^46^, respectively. 1000 replicates were used for Bootstrap analyses and only bootstraps above 50% were at the branch nodes of the phylogenetic tree^47^. The out-group taxon used during the construction of the phylogenetic tree for the present study was the *Bacillus subtilis* strain.

### Data analysis

All the sampling point measurements of (physical-chemical parameters tests) carried out in the present study were duplicates^48^. Calculations of mean and standard deviation were also carried out using the Microsoft (MS) Excel 2010 spreadsheet for each sampling point. Pearson’s correlation coefficient analysis was used to measure aspects of the linear relationship between various physicochemical parameters observed in all water samples^49,50^.

## Results

### Physical and chemical parameters

The TDS, pH, DO, and salinity from water samples showed values that met the recommended standards for domestic requirements as established by the World Health Organization (WHO). The most contrasting difference between the water containers was that the turbidity of the SD water samples was almost 20 times as high as that of the SM water samples. The Turbidity in SM samples ranged between 1 and 2.2 NTU, whereas in the SD samples range was between 17 and 22.3 NTU and this was because of the biofilm that was dislodged from the inner walls of the container through shaking. The water conditions (Table S2) at the time of samplings provided information about the environmental conditions of the microalgae in their habitat, which were partially favourable for their growth.

### Correlation analysis between physicochemical properties of water samples

Correlation (r) analysis (Table 3) was used to understand the relationship between the physicochemical parameters of the water samples. Analysis of seven physicochemical parameters using the Pearson correlation analysis shows that there was a strong correlation between EC and TDS (r=0.820), Salinity and DO (r=0.860) and Temperature and DO (*r*=0.742) at a 0.01 level of significance. Moderate positive correlations were observed between salinity and turbidity (*r*=0.668) and temperature and salinity (r=0.592). Negative correlations were revealed between DO and TDS (*r*=−9.55), Salinity and TDS (=−0.668), Temperature and TDS (*r*=−0.725), DO and EC (=−0.697), temperature and EC (*r*=−0.872), pH and turbidity (=−0.860), and salinity and pH (=−0.825).

**Table 3:** The Pearson’s correlation analysis between physicochemical properties of water samples.

	TDS	EC	Turbidity	DO	pH	Salinity	Temperature
TDS	1						
EC	0.820**	1					
Turbidity	− 0.206	0.373*	1				
DO	− 0.955	− 0.697	0.400*	1			
pH	0.160	− 0.268	− 0.860	− 0.436	1		
Salinity	− 0.668	− 0.317	0.668**	0.858**	− 0.825	1	
Temperature	− 0.725	− 0.872	− 0.198	0.742**	− 0.118	0.592**	1

*TDS total dissolved oxygen, *EC* electrical conductivity, *DO* dissolved oxygen.

**Correlation is significant at the 0.01 level (2-tailed), *Correlation is significant at the 0.01 level (2-tailed).

### Morphological characterization

**Figure 3:**
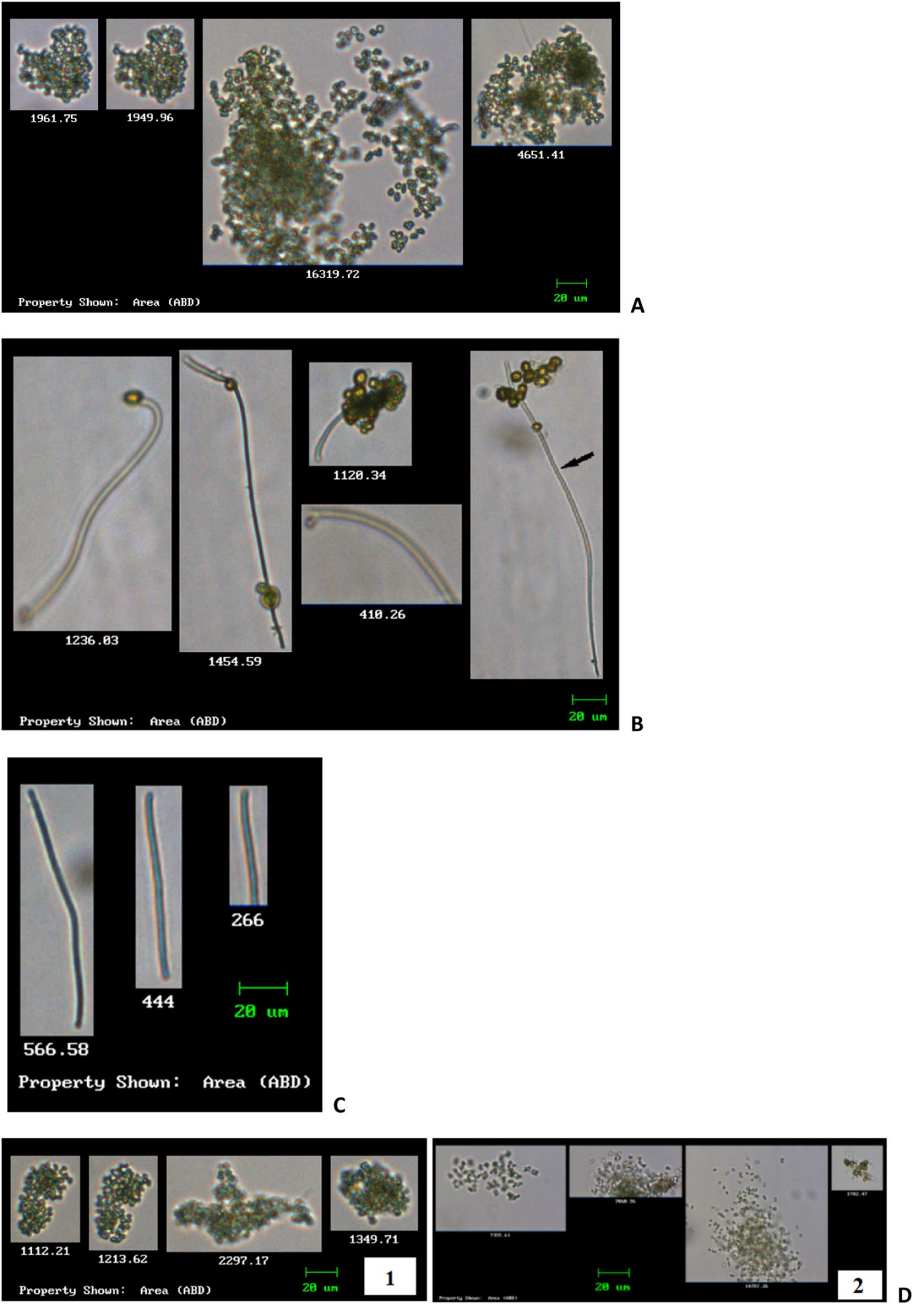
Representative FlowCAM images of genus *Microcystis* sp. (**A**) obtained from the SD-2 water sample; *Leptolyngbya* sp. (**B**) and *Phormidium* sp. (**C**) obtained from the SD-1 water sample; and *Microcystis* sp. (**D1-2**) obtained from the SM-1 and SM-2 water samples, respectively. All images are < 20 µm in diameter.

Images recorded during analyses reported the appropriate recognition of individual cyanobacteria species with an occasional appearance of other contamination (i.e. unidentified particles) and images containing more than one type of species (Figure 3B). However, the ratio of contamination and misrecognized images represented less than 15% of the total images. Although the sharpness of other cyanobacterial species in some images was affected by a wide range of focusing within the 100 µm depth of the flow cell, their shape was still visually recognizable.

Each analysis of a 1.00 mL sample provided between 2000 and 4000 images, of which identification of cyanobacterial species was possible. The images obtained from the sample SD-1 analysis showed cyanobacterial species, debris and other materials. A cyanobacterial species was observed in sample SD-1. The isolated morphological characteristics resemble those of the genus *Phormidium* sp. (Figure 3C). *Phormidium* sp. is a cyanobacterial species that belong to the order (Oscillatoriales) and is filamentous nature^51^. According to Palinska et al.^52^, *Phormidium* sp. is characterized based on morphological features such as thin, hyaline, partly diffluent, or completely dissolved sheaths, which cause filaments to stick together in mat–like layers or structures that may contribute to biofilm formation^53^. This unbranched filamentous species does not produce any specialized cells, such as heterocysts. Individual cells of *Phormidium* sp. are between 2.8 and 3.5 µm wide and 5 to 8 µm in length, while filaments extend up to 400 µm^53^. Another genus that was recognized based on morphological characteristics was *Leptolyngbya* sp. (Figure 3B)*.* Images of *Leptolyngbya* sp. were captured in sample SD-1. Prihantini et al.^54^ reported that *Leptolyngbya* is one of the cyanobacteria that grow well in culturing and they are fine filaments that are long and thin, with 0.5 to 3.2 μm dimension. They have heterocysts^54^.

A total of 4554 photo images were obtained from the water sample SD-2. Characteristics and morphological features of the dominant isolate obtained from this sample have demonstrated close similarity with the genus *Microcystis* sp. (Figure 3A). The individual cells of the colonies were in the range of 10 µm. Cells are green in colour and spherical. The cell diameter varies from 0.5 to 9 µm, which then classifies the strains as large type. The cells are grouped tightly or sparsely within fine, colourless colonial mucilage which is not seen in preserved material. Each colony of *Microcystis* consists of thousands of very small individual cells that are spherical to sub-spherical without individual mucilage sheaths. The cells appear a pale blue-green colour and often appear black when viewed through a light microscope and this is because of vacuoles that are located within the cells. The gas vacuoles allow the colony to drift through water layers to find the optimal amount of sunlight. Similar species of the genus *Microcystis* was also captured in sample SM-1 and SM-2 as shown in Figure 3D.

### Molecular technique to confirm the identity of cyanobacteria species

#### Isolation and identification of cyanobacteria

**Figure 4:**
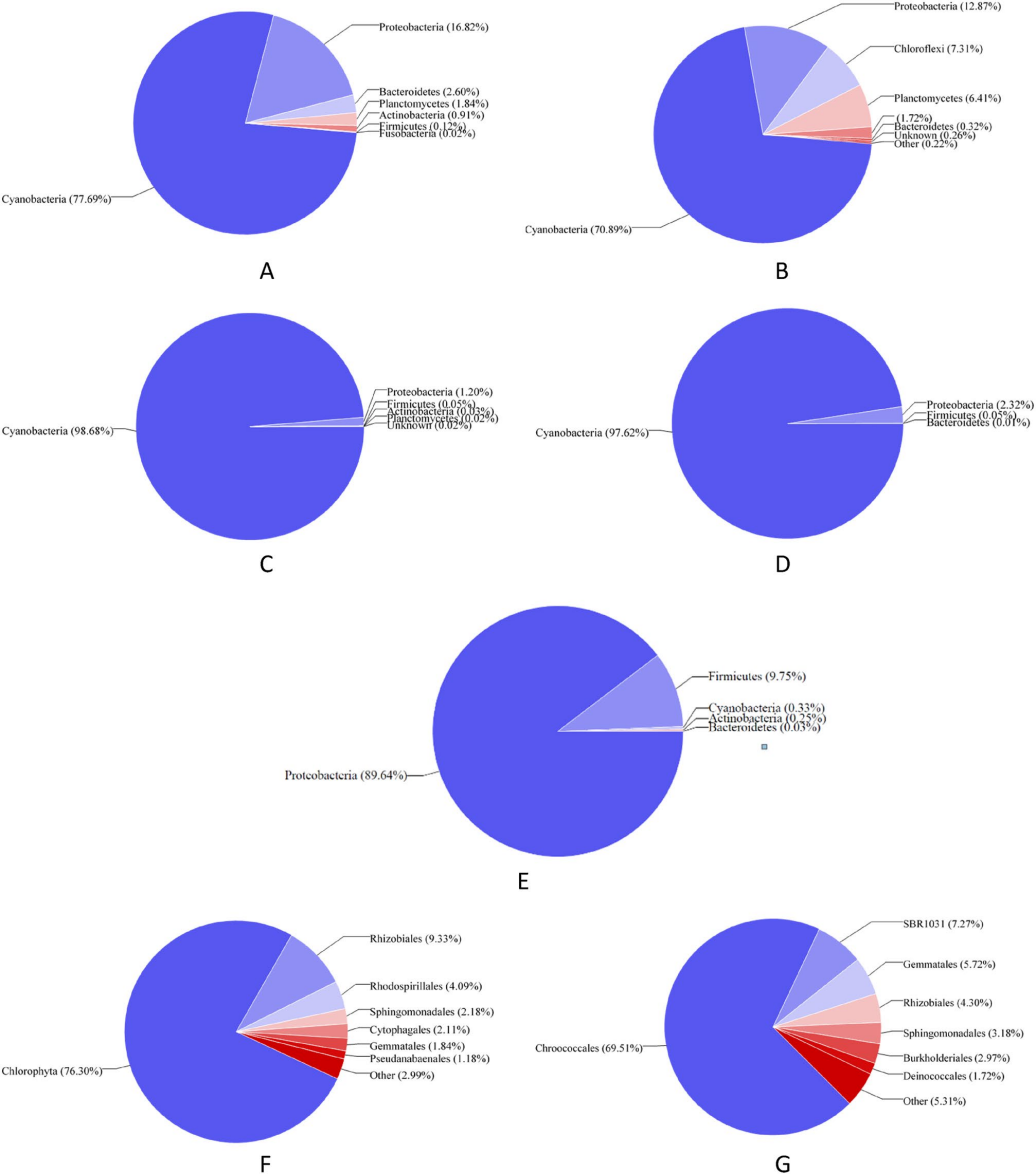
Pie charts showing the relative proportion of phyla: (**A**) (GFF1), (**B**) (GFF2), (**C**) (P1), (**D**) (P2), and (**E**) (P3); and order: (**F**) (GFF1) and **G** (GFF2) amplified by the primers set used in the present study.

The results from the analyses (shown in Figure 4) indicated that the primers set adopted in the present study targeting the 16S rRNA genes were able to target the cyanobacterial sequences. The analysis further showed that cyanobacterial sequences formed 77.6 %, 70.8 %, 98.6 %, 97.6 % and 0.33 % of total sequences amplified by 27F27F and 14292R primers in samples GFF1, GFF2, P1, P2, and P3, respectively. Therefore, these showed that the phylum cyanobacteria were found to have the largest fractions of the predicted target sequences in all the collected samples except for sample P3. The analyses of the present study further indicated that the primer pairs showed higher taxonomic specificity by targeting a wide range of phyla available in the National Center for Biotechnology Information (NCBI) database (Figure 4A–E). In sample GFF1, about 7 main phyla were amplified; whereas, in sample GFF2, P1, P2, and P3, the main phyla, which were amplified were 8, 6, 4 and 5, respectively.

The present study also considered the distribution of cyanobacterial morphotypes, where more than 8 genera were detected in all the cultured samples, representing the order Oscillitoriales, Chroococcales, Pseudanabaenales, Chroococcidiopsidales and Synechococcales. Genus *Aphanothece, Leptolyngbya, Microcystis* and *Chroococcus* amongst others listed in Table S3 were the most genera observed in all the samples. In samples P1 and P2, about 98.7 and 97.6 % of unclassified genera of cyanobacteria were observed, respectively. From the results obtained (Figure 4F–G), the highest diversity of cyanobacteria was found in cultured sample G (GFF2), where order Chroococcales had the largest fraction; followed by sample F (GFF1), where order Pseudanabaenales was found with a fraction of 1.18 %. Analysis of samples further revealed that sequences of *Leptolyngbya* and *Aphanothece* dominated sample GFF1, with *Microcystis* dominating sample GFF2. Table 4 shows that most of the species found have the potential to produce various types of cyanotoxins. According to Table 4, all the cyanobacterial species listed are capable of producing MCs. Based on these results, the order Chroococcales cyanobacteria could be considered a versatile species since they were distributed throughout the cultured water samples. Based on 16S rRNA gene sequences and their morphological features, 9 isolates were identified up to species level, 8 isolates up to genus level, and 5 isolates up to order level.

**Table 4:** Toxic cyanobacterial species and their potential cyanotoxins.

Strain	MCs	CYN	STX	ANT	BMAA	NOD	Reference
*Stanieria cyanosphaera* sp.	*						^55^
*Chroococcidiopsis* sp.	*				+		^56^
*Gomphosphaeria* sp.	*						^57^
*Oscillatoria* sp.	*	+	*	+	*		^58–60^
*Leptolyngbya* sp.	*						^55,61,62^
*Pseudanabaena* sp.	*		*	+			^55,63–65^
*Nostoc* sp.	*					*	^55,65,66^
*Phormidium* sp.	*		+	+			^65,67^
*Chroococcus* sp.	*		+	*			^64^
*Microcystis* sp.	*			*			^2,30,68^
*Eucapsis* sp*.*	*						^69^

*MCs microcystins, *CYN* Cylindrospermopsin, *SXT* saxitoxin, *ANT* anatoxin-a, *BMAA* β-N-methylamino-L-alanine, *NOD* nodularin.

From these observations, it was found that samples GFF1 and GFF2 appear to have the same cyanobacterial sequences as represented by the sequences (Figure 4A–B and Figure 4F–G). Similarly, samples P1 and P2 also appear to have the same cyanobacterial sequences as represented by the sequences (Figure 4C–D). Sample P3 had the lowest cyanobacterial sequences compared to other samples. Water samples from GFF1 and GFF2 were used to soften the maize seeds during the grinding process. However, none of the cyanobacterial species observed in GFF1 and GFF2 was detected in samples P2 and P3. Nonetheless, only unknown cyanobacterial order and species were observed in samples P1 and P2. Therefore, it was assumed from these results that the grinding process (temperatures of the grinding machines, shown in Table S4) affects the viability of cyanobacterial species. In sample P3, there was almost an absence of cyanobacterial species (0.33% of cyanobacterial sequences). It was assumed that probably the high temperatures/heat of cooking the porridge led to the death and destruction of the cyanobacterial species. The temperature during sample preparation (cooking) was above 92 °C.

**Figure 5:**
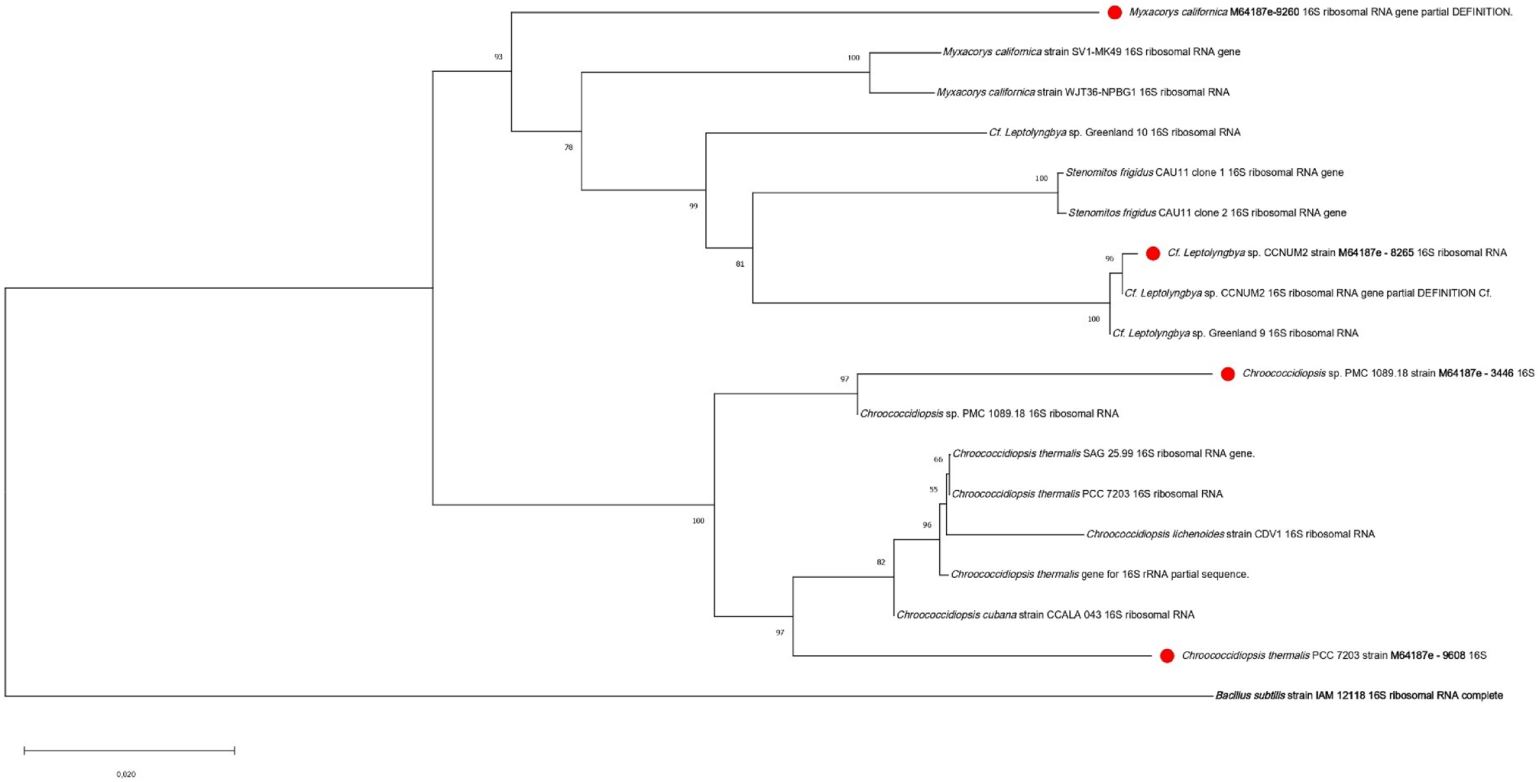
Topology of the 16S rRNA gene sequences of the cyanobacterial strains isolated from the present study and their closely related sequences from the NCBI database constructed using the neighbor-joining method^46^. The percentage of replicate trees in which the associated taxa clustered together in the bootstrap test (1000 replicates) are shown next to the branches^47^. Five base substitutions for nucleotide positions are represented by the scale bar. The sequences obtained in the present study are indicated by red rhombus.

Table 5 shows some of the cyanobacterial strains that were identified using molecular characters. This was done as some of the cyanobacteria could not be identified using morphological characters and was performed after the isolation and culturing of samples. Based on the 16S rDNA sequence homology search by the BLAST program, Table 5 also shows cyanobacterial strains with their closest related sequences obtained from the NCBI database. Strain m64187e-8265 was closely related to *Cf. Leptolyngbya* sp. CCNUM2 with 100% similarity; m64187e-5125 was closely related to *Chroococcus* sp. FPU101 with a similarity of 93%. A similarity of 97% similarity was found between m64187e-3446 and *Chroococcidiopsis* sp. PMC 1089.18. Another close similarity of 93% was found between the cyanobacterial species *Microcystis ichthyoblabe* gene and strain m64187e-6855. Furthermore, strain m64187e-7833 had 98% similarity with cyanobacterial species *Eucapsis* sp*.* 091 and 93% similarity was observed between strain m64187e-9608 and cyanobacterial species *Chroococcidiopsis thermalis* PCC 7203. Lastly, a similarity of 92 % was found between strain m64187e-9260 and *Myxacorys californica* strain SV1-MK49. Figure 5 shows the phylogenetic position of strains obtained in the present study among their closely related cyanobacterial species. Sequences obtained in the present study are indicated by a black dot and an outgroup taxon is shown in bold.

**Table 5:** Blast search results for cyanobacterial strains used in the present study based on 16S rRNA sequence data.

Strain	Closest species	Accession number	Identity (% homology)
m64187e-8265	*Cf. Leptolyngbya* sp. CCNUM2	MN544286.1	100
m64187e-5125	*Chroococcus* sp. FPU101	LC229082.1	93
m64187e-6855	*Microcystis ichthyoblabe* gene for 16S	AB023280.1	93
m64187e-3446	*Chroococcidiopsis* sp. PMC 1089.18	MW405089.1	97
m64187e-7883	*Eucapsis* sp. 091	KX151869.1	98
m64187e-9608	*Chroococcidiopsis thermalis* PCC 7203	NR_102464.1	93
m64187e-9260	*Myxacorys californica strain SV1-MK49*	AY239605.1	92

## Discussion

The work presented here was initiated to assess the diversity of the cyanobacterial composition of the process water, maize meal, and porridge samples using the FlowCAM-based imaging flow cytometry together with the PCR. Images of cyanobacteria species were captured using the FlowCAM and morphological characterization was performed based on available literature. FlowCAM observations revealed the presence of the genus *Microcystis*, *Phormidium* and genus *Leptolyngbya* in water samples. The most dominant cyanobacterial genera were the *Microcystis* species in sample SD-2. The diversity of cyanobacterial species in the present study showed comparable observations to previous studies conducted by Park et al.^35^, Patel et al.^70^ and Mirasbekov et al.^68^. Therefore, the present study further confirmed that FlowCAM can be used to rapidly process, screen and accurately detect a large volume of water samples. This study was able to directly detect cyanobacteria species in real time without staining or lysis. This is compatible with what has been reported by Park et al.^35^, who further highlighted that the FlowCAM allows early preventive measurements to be implemented. Furthermore, the PCR-based approach was conducted as a complementary method to confirm the flow cytometric analysis results. Identification using molecular characters was needed to identify cyanobacteria that could not be identified by morphological characters^54^. About seven phyla were amplified, of which the cyanobacteria had the largest fractions in most of the samples. Similar results were obtained in a study conducted by Wanigatunge et al.^37^ who observed from cultured samples, cyanobacterial genera from order Oscillitoriales and Chroococcales, amongst others that they observed. Though Wanigatunge et al.^37^ had recorded 24 cyanobacterial genera, out of those 24 only a few similar genera could be observed in the present study. However, a few extra genera namely *Eucapsis, Myxacorys* and *Chalicogloea* were identified, which were not observed in the study by Wanigatunge et al.^37^.

This study observed the effects of the grinding machines and temperature on the viability of cyanobacterial species. Cyanobacterial species detected in samples GFF1 and GFF2 were not detected in samples P1 and P2. This implies that different temperatures or extremely warm air (Table S4) within the grinding machines probably killed cyanobacterial species. Gallina et al.^71^ reported that a loss of diversity could be expected for the cyanobacteria community when exposed to air with hot temperatures. Similarly, sample P3 had a very low presence of cyanobacterial species compared to P2 (used to prepare P3) as this sample was subjected to high temperatures (Table S4) during sample preparation (cooking). O’Keeffe^72^ reported that cyanobacteria die at high temperatures. At boiling point, cells of cyanobacteria burst and cyanotoxins are released into the water and may increase toxin levels^72^. Sklenar et al.^73^ and Spoof et al.^74^ reported that MCs do not degrade or break down during boiling. Morais et al.^75^also reported in their study that boiling does not significantly change the MCs content after being boiled at 100 °C.

In addition, the present study further utilized the BLAST program (NCBI) to search for cyanobacterial strains that are closely related to the strains obtained in this study. The BLAST search revealed that the strains obtained in the present study were closely related to potentially cyanotoxins-producing species of cyanobacteria such as *Microcystis ichthyoblabe, Chroococcus, Eucapsis* and *Chroociccidiopsis*^30,56,64,68,69^. Furthermore, sequences of cyanobacterial species observed in the present study were used for the phylogenetic analysis. The phylogenetic tree constructed based on 16S rRNA showed the phylogenetic position of the strains among their closely related cyanobacteria (Fig. 5). According to the phylogenetic tree (Fig. 5) based upon its 16S rRNA, strain M64187e-3446 (hereafter referred to as *Chroococcidiopsis* sp. PMC 1089.18 strain M64187e-3446 16S) was closely positioned with *Chroococcidiopsis* sp. PMC 1089. 18 16S ribosomal RNA strain isolated from the museum in France^76^; strain m64187e-8265 (hereafter referred to as *Cf. Leptolyngbya* sp. CCNUM2 strain M64187e-8265 16S) was closely positioned with *Cf. Leptolyngbya* sp. CCNUM2, isolated in China^77^ with accession number MN544286.1; strain m64187e-9260 (hereafter referred to as *Myxacorys californica* M64187e-9260 16S ribosomal RNA gene partial DEFINITION) from order Synechococcales was positioned close to strain *Myxacorys californica* strain SV1-MK49 isolated from a stone monument in central Mexico^78^. In Figure S3, strain M64187e-0843 (hereafter referred to as *Aphanothece hegewaldii* M64187e-0843 16S) was positioned close to *Aphanothece hegewaldii* SAG 253.80 16S with accession number KM019993, isolated in Germany^79^. Another strain (M64187e-0123 16S, hereafter referred to as *Chalicogloea* sp. M64187e-0123 16S) from order Chroococcales was positioned close to *Chalicogloea cavernicola* CCALA 975 16S with accession number JQ967037, isolated in Spain^80^ (Figure S4). Strain m64187e-5125 (hereafter referred to as *Chroococcus* sp. M64187e-5125 16S) from the order Chroococcules was positioned close to strain *Chroococcus* sp. FPU101, isolated from Japan^81^ with accession number LC229082.1 (Figure S5). Another close similarity amongst others from the phylogenetic tree was between strain M64187e-7883 (hereafter referred to as *Eucapsis* sp. M64187e-7883 16S ribosomal RNA gene partial sequence) and strain *Eucapsis* sp. 019 16S (Figure S6) with accession number KX151869, isolated in Czech Republic. Lastly, Strain M64187e-6855 (hereafter referred to as *Microcystis ichthyoblabe* M64187e-6855 16S) as shown in Figure S7 was found positioned closed to *Microcystis ichthyoblabe* gene for 16S rRNA partial sequence with accession number AB023280, isolated from Japan^82^. The morphology and phylogenetic position of the isolated cyanobacterial strains in the present study were well supported by the previous research^83^. Therefore, these results confirmed the importance and usefulness of the 16S rRNA gene as a valuable tool for the identification of cyanobacteria up to order or genus level. This study provides results that will contribute greatly to the knowledge of cyanobacterial diversity in water supplies used for food processing in terms of morphology and their phylogeny based on 16S rRNA sequences.

## Conclusion

The study aimed to evaluate the presence of cyanobacteria in processed water, uncooked maize meal and cooked maize meal (porridge). In the present study, the use of a slow flow rate in the FlowCAM was adopted, which, however, optimized images of good quality as recommended by previous studies. The FlowCAM-based method enabled the present study to capture thousands of images that were then identified using their morphological characteristics. Cyanobacterial species from order Nostocales, Pseudanabaenales, Oscillatoriales, and order Chroococcales, which all have the potential to produce cyanotoxins were amplified and identified in process water, raw maize meal and porridge samples using PCR. The most dominant cyanobacteria species was the genus *Microcystis* captured in samples SM1, SM2 and sample SD2. The other species detected and identified using molecular techniques were the genus *Leptolyngbya* and the genus *Aphanothece* in sample SD1. As such, the presence of genera *Microcystis, Leptolyngbya, Phormidium, and Chroococcus* amongst the other genera found in water from the containers can pose threat to human health as these genera have been reported to release cyanotoxins such as MCs, CYNs. The distribution of cyanobacteria in water samples was different compared to that of maize samples. Also, different distribution was observed between maize samples and porridge samples. This was influenced by the temperatures of the grinding machines and the temperatures of the cooking stove during sample preparation. It was concluded in the present study that high temperatures/heat affect the occurrence of cyanobacteria while increasing the levels of cyanotoxins as these toxins are released when cells of cyanobacteria lysis at higher temperatures. The presence of cyanotoxins in water and porridge may lead to a range of potential effects on human health. The present study also revealed that cyanobacteria in water containers are from the water source where water is collected and could have grown inside the containers as these containers are light permitting. It was also proven in this study that the presence of cyanobacteria in water containers could be an important route of human exposure to cyanotoxins for the reason that such water can contaminate food (maize meal) during processing. Therefore, the present study recommends the use of dark colour containers for the collection and storage of water for use. Dark containers inhibit light penetration and therefore limit cyanobacteria proliferation in stored water. The present study proved that the FlowCAM and PCR can be used for cyanobacterial identification and also demonstrated that these instruments can be used for proper management of water quality in freshwater systems and to provide public water that is safe for domestic use. This study successfully detected and identified potentially toxic cyanobacteria species. However, levels of cyanotoxins released by these species were not quantified. Therefore, the present study suggests that future investigations should be focused on quantitative analysis of cyanotoxins in processed water, uncooked maize meal and cooked maize meal.

### Study limitations

Difficulties were encountered during sampling. Access to the sampling site was not an issue but collecting water samples and operating the machines became a problem for the owners. They did not understand what samples were for until we explained. Also, people (owner’s customers) who were there during sampling felt threatened, they thought what we are doing may contaminate their water and food. As a result of all these, we only sampled for one season instead of two seasons, which could have allowed the present study to be a comparative study between dry and wet seasons. The use of FlowCAM requires fine-tuning to get images with good or high resolution. This process is performed when the sample is injected and running and therefore you end up losing images of your target species.

## Supplementary Information

Supplementary Information 1.

Supplementary Information 2.

## Data Availability

The datasets generated during the current study are available in the National Centre for Biotechnology Information (NCBI) repository, Accession numbers: ON514589, ON514590, ON514592, OP323089, OP323093, OP323096, OP323098 and OP323105.

## References

[1] Jagals P, Fosso-Kenkeu E, du Preez H (2008). Exposure of rural households to toxic cyanobacteria in container-stored water. Water S. A..

[2] Almuhtaram H, Kibuye FA, Ajjampur S, Glover CM, Hofmann R, Gaget V, Owen C, Wert EC, Zamyadi A (2021). State of knowledge on early warning tools for cyanobacteria detection. Ecol. Ind..

[3] Mutoti M, Gumbo J, Jideani AIO (2022). Occurrence of cyanobacteria in water used for food production: A review. Phys. Chem. Earth Parts A B C.

[4] Zanchett G, Oliveira-Filho EC (2013). Cyanobacteria and cyanotoxins: from impacts on aquatic ecosystems and human health to anticarcinogenic effects. Toxins.

[5] Vaughan L, Zamyadi A, Ajjampur S, Almutaram H, Freguia S (2022). A review of microscopic cell imaging and neural network recognition for synergistic cyanobacteria identification and enumeration. Anal. Sci..

[6] Berry J (2013). Cyanobacterial toxins in food-webs: Implications for human and environmental health. InTech.

[7] Drobac D, Tokodi N, Simeunovic J, Baltic V, Stan D, Svircev Z (2013). Human exposure to cyanotoxins and their effects on health. Arh. Hyg. Rada. Toksikol..

[8] Miller A, Russell C, Wiens M (2017). Irrigating food crops with water containing cyanobacteria blooms. Environ. Health Rev..

[9] Swanepoel A, Du Preez HH, Cloete N (2017). The occurrence and removal of algae (including cyanobacteria) and their related organic compounds from source water in Vaalkop Dam with conventional and advanced drinking water treatment processes. Water S. A..

[10] Graham MD, Cook J, Graydon J, Kinniburgh D, Nelson H, Pilieci S, Vinebrooke RD (2018). High-resolution imaging particle analysis of freshwater cyanobacterial blooms. Limnol. Oceanogr. Methods..

[11] Reddy MRK, Mastan SA (2011). Algal toxins and their Impact on human Health. Biomed Pharmacol. J..

[12] Merel S, Villarin MC, Chung K, Snyder S (2013). Spatial and thematic distribution of research on cyanotoxins. Toxicon.

[13] Dai R, Wang P, Jia P, Zhang Y, Chu X, Wang Y (2016). A review on factors affecting microcystins production by algae in aquatic environments. World J. Microbiol. Biotechnol..

[14] Turner AD, Dhanji-Rapkova M, O’Neill A, Coates L, Lewis A, Lewis K (2018). Analysis of Microcystins in cyanobacterial blooms from freshwater bodies in England. Toxins..

[15] Welten RD, Meneely JP, Elliott CT (2020). A comparative review of the effect of Microcystin-LR on the proteome. Expo. Health.

[16] Boopathi T, Ki J (2014). Impact of environmental factors on the regulation of cyanotoxin production. Toxins.

[17] Association of Public Health Laboratories (APHL). Cyanotoxins: A Guidance Document for Public Health Laboratories. (2021). https://www.aphl.org/aboutAPHL/publications/Documents/EH_2021_Cyanotoxin_Guide.pdf (Accessed 2022/08/30).

[18] Mirasbekov Y, Abdimanova A, Sarkytbayev K, Samarkhanov K, Abilkas A, Potashnikova D, Arbuz G, Issayev Z, Vorobjev IA, Malashenkov DV, Barteneva NS (2021). Combining imaging flow cytometry and molecular biological methods to reveal presence of potentially toxic algae at the Ural river in Kazakhstan. Front. Mar. Sci..

[19] Codd GA, Bell S, Kaya K, Ward C, Beattie K, Metcalf J (1999). Cyanobacterial toxins, exposure routes and human health. Eur. J. Phycol..

[20] Kubickova B, Babica P, Hilscherova K, Sindlerova L (2019). Effects of cyanobacterial toxins on the human gastrointestinal tract and the mucosal innate immune system. Environ. Sci. Eur..

[21] Hilborn ED, Beasley VR (2015). One health and cyanobacteria in freshwater systems: Animal illnesses and deaths are sentinel events for human health risks. Toxins.

[22] Backer LC, Carmichael W, Kirkpatrick B, Williams C, Irvin M, Zhou Y, Johnson TB, Nierenberg K, Hill VR, Kieszak SM, Cheng YS (2008). Recreational exposure to low concentrations of *Microcystins* during an Algal bloom in a small lake. Mar. Drugs..

[23] Massey IY, Yang F, Ding Z, Yang S, Guo J, Tezi C, Al-Osman M, Kamegni RB, Zeng W (2018). Exposure routes and health effects of *Microcystins* on animals and humans: A mini-review. Toxicon.

[24] Svircev Z, Drobac D, Tokodi N, Mijovic B, Codd GA, Meriluoto J (2017). Toxicology of microcystins with reference to cases of human intoxications and epidemiological investigations of exposures to cyanobacteria and cyanotoxins. Arch Toxicol..

[25] Vidal F, Sedan D, D’Agostino D, Cavalieri ML, Mullen E, Varela MMP, Flores C, Caixach J, Andrinolo D (2017). Recreational exposure during Algal bloom in Carrasco beach, Uruguay: A liver failure case report. Toxins.

[26] Ilieva V, Kondeva-Burdina M, Georgieva T, Pavlova V (2019). Toxicity of cyanobacteria. Organotropy of cyanotoxins and toxicodynamics of cyanotoxins by species. Pharmacia.

[27] Azevedo SM, Carmichael W, Jochimsen EM, Eaglesham GK (2003). Human intoxication by microcystins during renal dialysis treatment in Caruaru-Brazil. Toxicology.

[28] Camoying MG, Yniguez AT (2016). FlowCAM optimization: Attaining good quality images for higher taxonomic classification resolution of natural phytoplankton samples. Limnol. Oceanogr. Methods..

[29] Moza MI, Postolache C (2021). Optimized Protocol for Cyanobacterial 16S rRNA Analysis in Danube Delta Lakes. bioRxiv..

[30] Valadez-Cano C, Hawkes K, Calvaruso R, Reyes-Prieto A, Lawrence J (2022). Amplicon-based and metagenomic approaches provide insights into toxigenic potential in understudied Atlantic Canadian lakes. Facets..

[31] Sieracki CK, Sieracki ME, Yentsch CS (1998). An imaging-in-flow system for automated analysis of marine microplankton. Mar. Ecol. Prog. Ser..

[32] Lehman PW, Marr K, Boyer GL, Acuna S, Teh SJ (2013). Long-term trends and casual factors associated with *Microcystis* abundance and toxicity in San Francisco Estuary and implications for climate change impacts. Hydrobiologia.

[33] Poulton NJ, Barteneva NS, Vorobjev IA (2016). FlowCAM: Quantification and classification of phytoplankton by imaging flow cytometry. Imaging Flow Cytometry: Methods and Protocols, Methods in Molecular Biology.

[34] Spaulding SA, Jewson DH, Bixby RJ, Nelson H, McKnight DM (2012). Automated measurement of diatom size. Limnol. Oceanogr. Methods..

[35] Park J, Kim Y, Kim M, Lee WH (2019). A novel method for cell counting of *Microcystis* colonies in water resources using a digital imaging flow cytometer and microscope. Environ. Eng. Res..

[36] Keshari N, Adhikary SP (2013). Characterization of cyanobacteria isolated from biofilms on stone monuments at Santiniketan. India Biofouling.

[37] Wanigatunge RP, Magana-Arachchi DN, Chandrasekharan NV, Kulasooriya SA (2014). Genetic diversity and molecular phylogeny of cyanobacteria from Sri Lanka based on 16S rRNA gene. Environ. Eng. Research.

[38] Lee E, Khurana MS, Whiteley AS, Monis PT, Bath A, Gordon C, Ryan UM, Paparini A (2017). Novel primer sets for next-generation sequencing-based analyses of water quality. PLoS ONE.

[39] Gofton AW, Oskam CL, Lo N, Beninati T, Wei H, McCarl V, Murray DC, Paparini A, Greay TL, Holmes AJ, Bunce M (2015). Inhibition of the endosymbiont “Candidatus Midichloria mitochondrii” during 16S rRNA gene profiling reveals potential pathogens in Ixodes ticks from Australia. Parasit. Vectors.

[40] Bose, P. Rapidly Identifying Harmful Blue-Green Algae with Mass Spectroscopy. AZoOptics, (2021). Accessed 17/09/2022. https://www.azooptics.com/Article.aspx?ArticleID=2089.

[41] van Vuuren, S. J., Taylor, J., Gerber, A. & van Ginkel, C. Easy identification of the most common freshwater algae. A guide for the identification of microscopic algae in South African freshwater. ISBN: 0–621–35471–6 (2006).

[42] Owen BM, Hallett CS, Cosgrove JJ, Tweedley JR, Moheimani NR (2022). Reporting of methods for automated devices: A systematic review and recommendation for studies using FlowCam for phytoplankton. Limnol. Oceanogr. Methods.

[43] Gumbo JR, Ross G, Cloete TE (2010). The isolation and identification of predatory bacteria from a Microcystis algal bloom. Afr. J. Biotechnol..

[44] Altschul SF, Madden TL, Schäffer AA (1997). Gapped blast and PSI-blast: A new generation of protein database search programs. Nucleic Acids Res..

[45] Nei M, Kumar S (2000). Molecular Evolution and Phylogenetics.

[46] Saitou N, Nei M (1987). The neighbor-joining method: A new method for reconstructing phylogenetic trees. Mol. Biol. Evol..

[47] Felsenstein J (1985). Confidence limits on phylogenies: An approach using the bootstrap. Evolution.

[48] Magonono M, Oberholster PJ, Shonhai A, Makumire S, Gumbo JR (2018). The presence of toxic and non-toxic cyanobacteria in the sediments of the Limpopo River Basin: Implications for human health. Toxins..

[49] Ouattara M, Zongo F, Zongo B (2021). Species diversity of cyanobacteria and desmids of a drinking water source under anthropogenic pressure, and their implication in toxin production and water quality in Sub-Saharan Africa (Burkina Faso, Western Africa). J. Water Resour. Prot..

[50] Sankaranarayanan A, Poyil MM, Karuppiah P, Mohideen AP (2021). Effect of physico-chemical parameters on the population diversity of potentially harmful micro-algae during post-monsoon season along the Malabar coast. J. Pure Appl. Microbiol..

[51] Hotos GN (2021). Culture growth of the cyanobacterium *Phormidium sp*. in various salinity and light regimes and their influence on its phycocyanin and other pigments content. J. Mar. Sci. Eng..

[52] Palinska KA, Deventer B, Hariri K, Lotocka M (2011). A taxonomic study on *Phormidium*–group (cyanobacteria) based on morphology, pigments, RAPD molecular markers and RFLP analysis of the 16S rRNA gene fragment. Fottea.

[53] Koch M, Noonan A, Qiu Y, Dofher K, Kieft B, Mottahedeh S, Shastri M, Hallam S (2022). The survivor strain: isolation and characterization of *Phormidium yuhuli* AB48, a filamentous phototactic cyanobacterium with biotechnological potential. Front. bioeng. Biotechnol..

[54] Prihantini, N.B., Sjamsuridzal, W. & Yokota, A., October. Characterization of culturable cyanobacteria isolated from geyser of Cisolok in West Java, Indonesia. In *AIP Conference Proceedings* (Vol. 2023, No. 1, p. 020135). AIP Publishing LLC (2018).

[55] Cordeiro R, Azevedo J, Luz R, Vasconcelos V, Gonçalves V, Fonseca A (2021). Cyanotoxin screening in BACA culture collection: Identification of new Cylindrospermopsin producing cyanobacteria. Toxins.

[56] Cox PA, Banack SA, Murch SJ, Rasmussen U, Tien G, Bidigare RR, Bergman B (2005). Diverse taxa of cyanobacteria produce -N-methylamino-L-alanine, a neurotoxic amino acid. Proc. Natl. Acad. Sci..

[57] Mooney KM, Hamilton JTG, Floyd SD, Foy RH, Elliott CT (2011). Initial studies on the occurrence of cyanobacteria and microcystins in Irish lakes. Environ. Toxicol..

[58] Christoffersen K, Kaas H (2010). Toxic cyanobacteria in water. A guide to their public health consequences, monitoring and management. Limnol. Oceanogr..

[59] Bernard C, Ballot A, Thomazeau S, Maloufi S, Furey A, Mankiewicz-Boczek J, Pawlik-Skowrońska B, Capelli C, Salmaso N, Meriluoto J, Spoof L, Codd GA (2016). Appendix 2: Cyanobacteria associated with the production of cyanotoxins. Handbook of Cyanobacterial Monitoring and Cyanotoxin Analysis.

[60] Mohamed ZA (2016). Breakthrough of *Oscillatoria limnetica* and microcystin toxins into drinking water treatment plants—Examples from the Nile river, Egypt. Water S. A..

[61] Frazao B, Martins R, Vasconcelos V (2010). Are known cyanotoxins involved in the toxicity of Picoplanktonic and Filamentous North Atlantic marine cyanobacteria?. Mar. Drugs.

[62] Somdee T, Kaewsan T, Somdee A (2013). Monitoring toxic cyanobacteria and cyanotoxins (microcystins and cylindrospermopsins) in four recreational reservoirs (Khon Kaen, Thailand). Environ. Monit. Assess..

[63] Borges HLF, Branco LHZ, Martins MD, Lima CS, Barbosa PT, Lira GAST, Molica RJR (2015). Cyanotoxin production and phylogeny of benthic cyanobacterial strains isolated from the northeast of Brazil. Harmful Algae.

[64] Wiltsie D, Schnetzer A, Green J, Vander Borgh M, Fensin E (2018). Algal blooms and Cyanotoxins in Jordan lake North California. Toxins.

[65] Graham JL, Dubrovsky NM, Foster GM, King LR, Loftin KA, Rosen BH, Stelzer EA (2020). Cyanotoxin occurrence in large rivers of the United States. Inland Waters.

[66] Ivanov D, Yaneva G, Potoroko I, Ivanova DG (2021). Contribution of Cyanotoxins to the ecotoxicological role of Lichens. Toxins.

[67] Wood SA, Puddick J, Fleming R, Heussner AH (2017). Detection of anatoxin-producing Phormidium in a New Zealand farm pond and an associated dog death. N. Z. J. Bot..

[68] Mirasbekov Y, Abdimanova A, Sarkytbayev K, Samarkhanov K, Abilkas A, Potashnikova D, Arbuz G, Issayev Z, Vorobjev IA, Malashenkov DV, Barteneva NS (2021). Combining imaging flow cytometry and molecular biological methods to reveal presence of potentially toxic algae at the Ural river in Kazakhstan. Front. Mar. Sci..

[69] Mioni C, Kudela R, Baxa D, Sullivan M, Hayashi K, Smythe UT, White C (2011). Harmful cyanobacteria blooms and their toxins in Clear Lake and the Sacramento-San Joaquin Delta (California). Delta Calif..

[70] Patel R, de Oliveira A, Newby R, Chu T (2019). Flow cytometric analysis of freshwater cyanobacteria: A case study. Water.

[71] Gallina N, Anneville O, Beniston M (2011). Impacts of extreme air temperatures on cyanobacteria in five deep peri-Alpine lakes. Limnol. Oceanogr..

[72] O’Keeffe, J. Cyanobacteria—Tapping into the risks to drinking water. (2019) https://ncceh.ca/content/blog/cyanobacteria-tapping-risks-drinking-water Accessed (21/09/2022).

[73] Sklenar K, Westrick J, Szlag D (2016). Managing cyanotoxins in drinking water: A technical guidance manual for drinking water professionals.

[74] Spoof L, Jaakkola S, Vazic T, Haggqvist K, Kirkkala T, Ventela AM, Kirkkala T, Svircev Z, Meriluoto J (2020). Elimination of cyanobacteria and microcystins in irrigation water—Effects of hydrogen peroxide treatment. Environ. Sci. Pollut. Res..

[75] Morais J, Augusto M, Carvalho AP, Vale M, Vasconcelos VM (2008). Cyanobacteria hepatotoxins, microcystins: Bioavailability in contaminated mussels exposed to different environmental conditions. Eur. Food Res. Technol..

[76] Hamlaoui S, Yéprémian C, Duval C, Marie B, Djédiat C, Piquet B, Bernard C, Duperron S (2022). The culture collection of cyanobacteria and microalgae at the French national museum of natural history: A century old but still alive and kicking! Including in memoriam: Professor Alain Couté. Cryptogam. Algol..

[77] Zhang ZC, Li ZK, Yin YC, Li Y, Jia Y, Chen M, Qiu BS (2019). Widespread occurrence and unexpected diversity of red-shifted chlorophyll producing cyanobacteria in humid subtropical forest ecosystems. Environ. Microbiol..

[78] Vazquez-Martinez J, Gutierrez-Villagomez JM, Fonseca-Garcia C, Ramirez-Chavez E, Mondragon-Sanchez ML, Partida-Martinez L, Johansen JR, Molina-Torres J (2018). Nodosilinea chupicuarensis sp. nov. (Leptolyngbyaceae, Synechococcales) a subaerial cyanobacterium isolated from a stone monument in central Mexico. Phytotaxa..

[79] Friedl, T., Hepperle, D. & van de Peer, Y. (2004) Documentation of characters of terrestrial algae in culture: R-DNA sequence analyses, morphology and cryo-preservation of reference strains. *German Program. Biodivers. Global Change*. p. 238.

[80] Roldan M, Ramirez M, Del Campo J, Hernandez-Marine M, Komarek J (2013). Chalicogloea cavernicola gen. nov., sp. nov. (Chroococcales, Cyanobacteria), from low-light aerophytic environments: Combined molecular, phenotypic and ecological criteria. Int. J. Syst. Evol. Microbiol..

[81] Ohki K, Kanesaki Y, Suzuki N, Okajima M, Kaneko T, Yoshikawa S (2019). Physiological properties and genetic analysis related to exopolysaccharide (EPS) production in the fresh-water unicellular cyanobacterium Aphanothece sacrum (Suizenji Nori). J. Gen. Appl. Microbiol..

[82] Kondo R, Kagiya G, Hiroishi S, Watanabe M (2000). Genetic typing of a bloom-forming cyanobacterial genus Microcystis in Japan using 16S rRNA gene sequence. Plankton Biol. Ecol..

[83] Cordeiro R, Luz R, Vasconcelos V, Gonçalves V, Fonseca A (2020). Cyanobacteria phylogenetic studies reveal evidence for polyphyletic genera from thermal and freshwater habitats. Diversity.

