# Supplementary figures and images for: Using FlowCam and molecular techniques to assess the diversity of Cyanobacteria species in water used for food production
Source: Sci Rep. 2022 Nov 8;12:18995. doi: 10.1038/s41598-022-23818-1 (PMC9643327; doi:10.1038/s41598-022-23818-1)

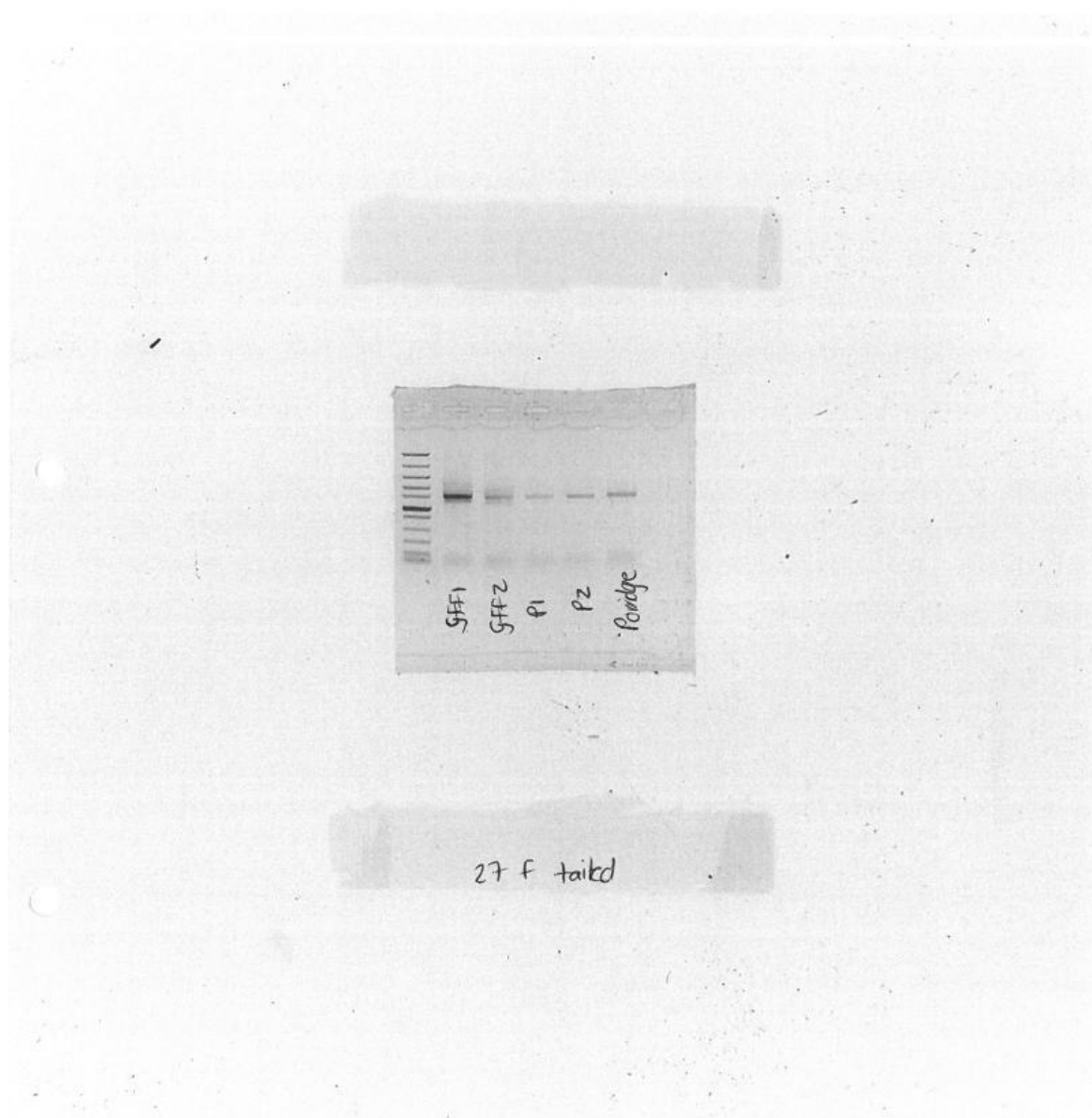

Mulalo  
FL 16S PCR  
02/07/2021  
# 022  
PacBio

**Figure XX:** Full-length gels (uncropped) from laboratory analysis.

Supplement: Supplementary file 1 — Supplementary Information 1. [file 41598_2022_23818_MOESM1_ESM.pdf]
