# Supplementary material for: Using FlowCam and molecular techniques to assess the diversity of Cyanobacteria species in water used for food production
Source: Sci Rep. 2022 Nov 8;12:18995. doi: 10.1038/s41598-022-23818-1 (PMC9643327; doi:10.1038/s41598-022-23818-1)
Supplement: Supplementary file 2 — Supplementary Information 2. [file 41598_2022_23818_MOESM2_ESM.pdf]

Using FlowCam and molecular techniques to assess the diversity of Cyanobacteria species in water used for food production

Mulalo I. Mutoti[1,*], Afam I. O. Jideani[2] and Jabulani R. Gumbo[1]

1. Supp. Tables

Run:	Mode: AutoImage<br>Priming Method: manual prime with sample<br>Flow Rate: 0.400 ml/min<br>Recalibrations: 0<br>Stop Reason: Sample Volume Processed<br>Sample Volume Aspirated: 0.9946 ml<br>Sample Volume Processed: 0.9700 ml<br>Fluid Volume Imaged: 0.1089 ml<br>Efficiency: 11.2%<br>Particle Count: 15077
Images:	Total: 2870<br>Used: 2868<br>Percentage Used: 99.93%<br>Particles Per Used Image: 5.26<br>Frame Rate: 19.73 fps<br>Intensity Mean: 151.82<br>Intensity Min: 150.71<br>Intensity Max: 153.38
Date/Time:	Start: 2020-12-09 08:55:23<br>End: 2020-12-09 08:57:57<br>Sampling Time: 00:02:25
Environment:	Software: VisualSpreadsheet 3.2.2<br>Magnification: 10X<br>Calibration Factor: 0.5563<br>DSP Firmware: 55<br>Serial No: 5049<br>Number of Processors: 2<br>Pump: C70 Syringe<br>Syringe Size: 5.00 ml

Table S1. An example of run summary and software settings employed in one of the analyses conducted in the study.

Sample	TDS (mg/L)	EC (mS/m)	Turbidity (NTU)	DO (mg/L)	pH	Salinity (ppm)	Temperature (^{0}C)
**SM-1**	37.8	52.6	2.17	7.68	7.8	23.8	20.1
**SM-2**	38.2	52.8	1.23	7.77	7.29	25.1	21.1
**SD-1**	38.4	56.3	22.31	7.54	6.84	25.5	19.5
**SD-2**	36.6	50.8	17.85	13.02	6.91	27.3	21.6

Table S2. Physical and chemical conditions of container water during sampling.

Toxicity	Order	Genus	Species
**Non-Toxic**	Chroococcales	*Chalicogloea*<br>Aphanothece	*Chalicogloea cavernicola*<br>*Aphanothece hegewaldii*
	Pseudanabaenales	*Myxacorys*	*Myxacorys californica*
**Toxic**	Chroococcales	*Chroococcus*<br>*Microcystis*	-<br>-
	Pseudanabaenales	*Leptolyngbya*	*Leptolyngbya boryana*
	Oscillatoriales	*Phormidium*	-
	Chroococcidiopsidales	*Chroococcidiopsis*	*Chroococcidiopsis thermalis*
	Synechococcales	*Eucapsis*	-

Table S3. Cyanobacteria detected by primers set in the present study.

Process	Temperature (^{0}C)	Processing Time (min)
Machine 1	59.8 – 66.0	5
Machine 2	21.1 – 25.4	6
Machine 3	28.9 – 29.8	5
Cooking stove and boiling water	89.5 – 92.7	20

Table S4. Thermal data for the grinding mill and cooking process.

2. Supp. Figures

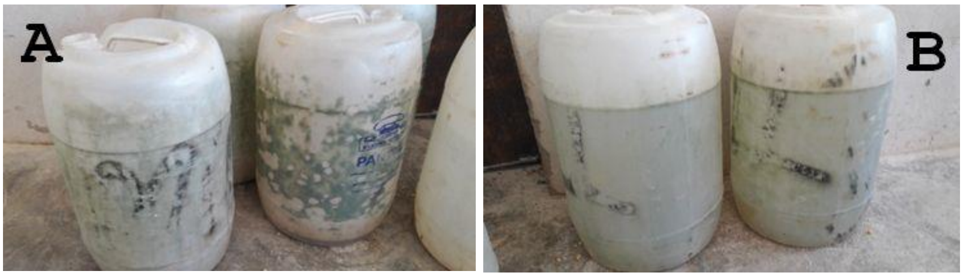

Figure S1. Biofilm on the sidewalls of containers used to collect and store process water.

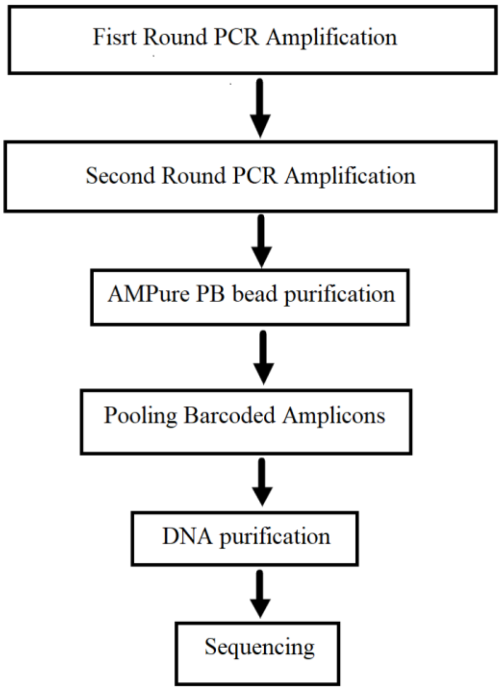

Figure S2. The general sequencing workflow adopted in the present study.

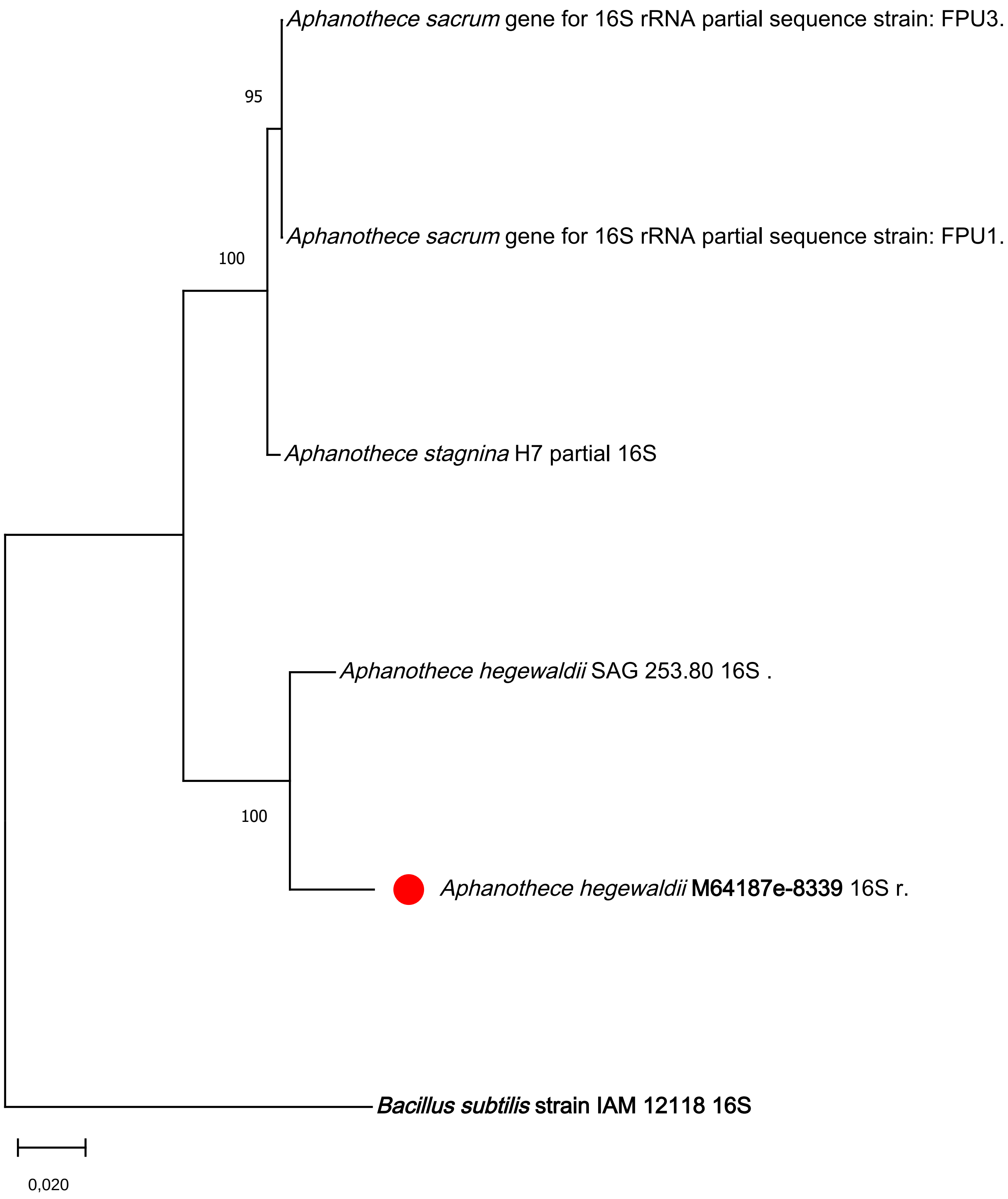

Figure S3. Topology of the 16S rRNA gene sequences of the *Aphanothece hegewaldii* sp. strain isolated from the present study and its closely related sequence from the NCBI database constructed using the neighbor-joining method[46]. The percentage of replicate trees in which the associated taxa clustered together in the bootstrap test (1000 replicates) are shown next to the branches[47]. Five base substitutions for nucleotide positions are represented by the scale bar. The sequences obtained in the present study are indicated by red dot.

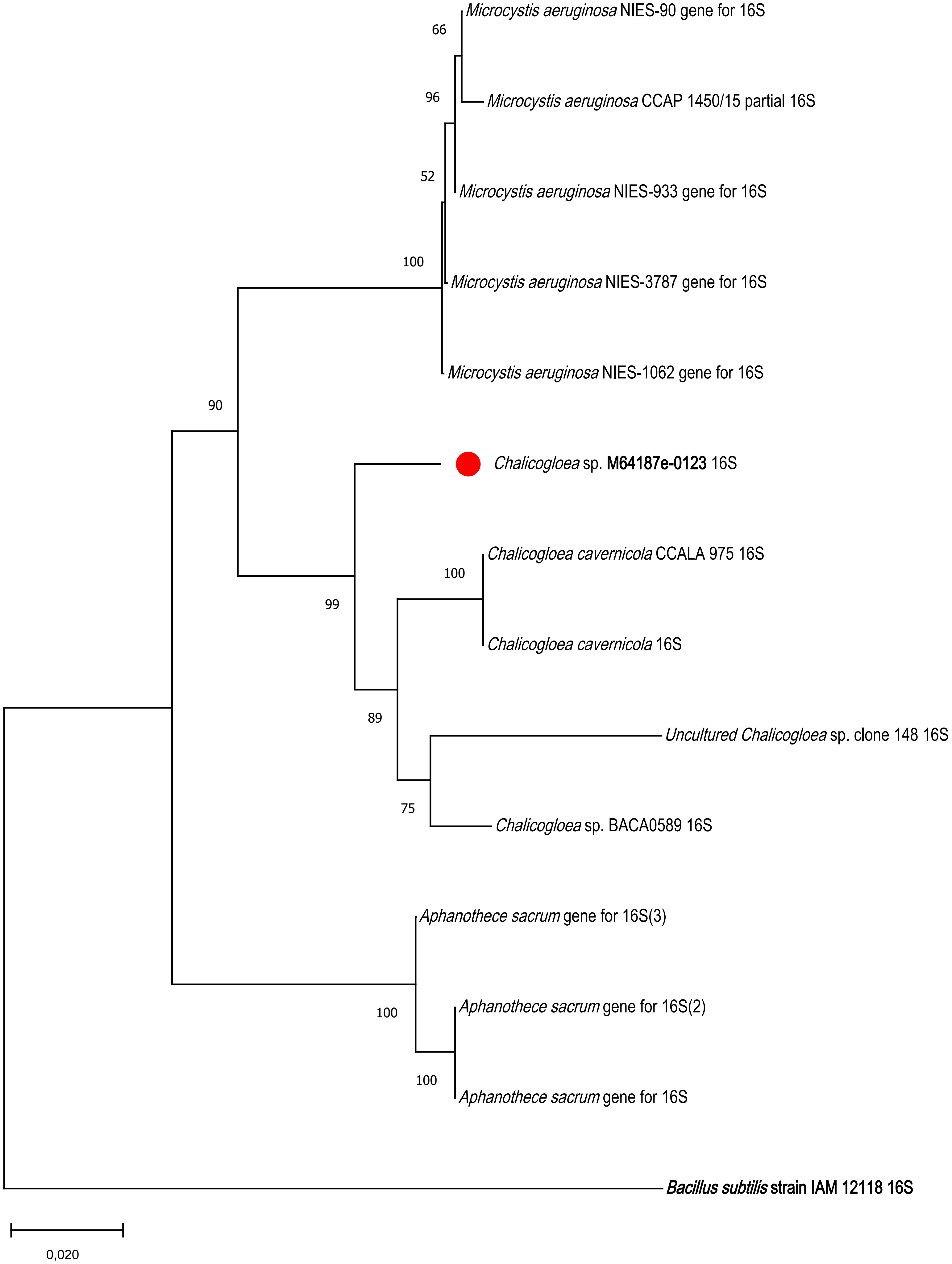

Figure S4. Topology of the 16S rRNA gene sequences of the cyanobacterial strain (*Chalicogloea* sp.) isolated from the present study and its closely related sequence from the NCBI database constructed using the neighbor-joining method[46]. The percentage of replicate trees in which the associated taxa clustered together in the bootstrap test (1000 replicates) are shown next to the branches[47]. Five base substitutions for nucleotide positions are represented by the scale bar. The sequences obtained in the present study are indicated by red dot.

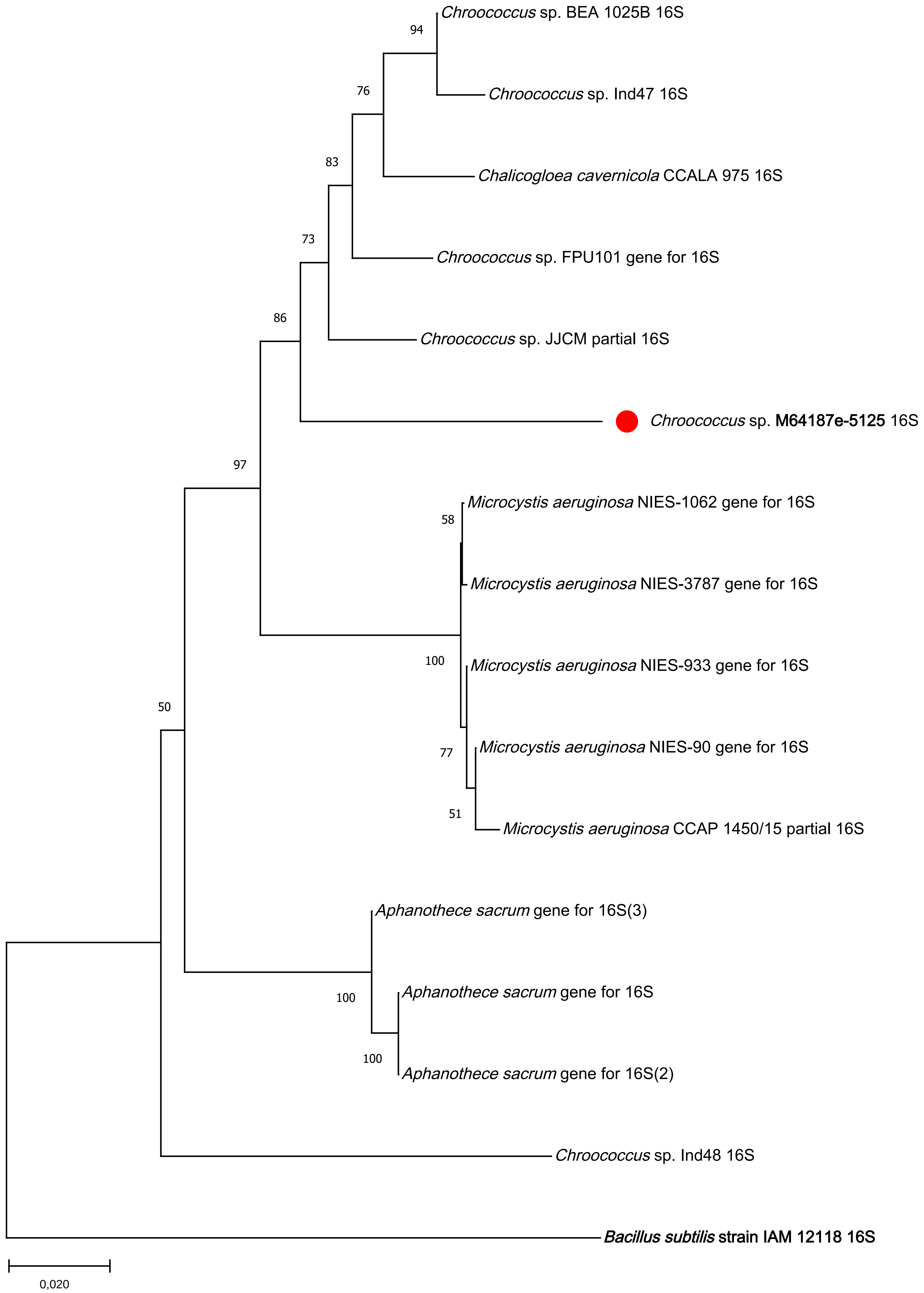

Figure S5. Topology of the 16S rRNA gene sequences of the cyanobacterial strain (*Chroococcus* sp.) isolated from the present study and its closely related sequences from the NCBI database constructed using the neighbor-joining method[46]. The percentage of replicate trees in which the associated taxa clustered together in the bootstrap test (1000 replicates) are shown next to the branches[47]. Five base substitutions for nucleotide positions are represented by the scale bar. The sequences obtained in the present study are indicated by red dot.

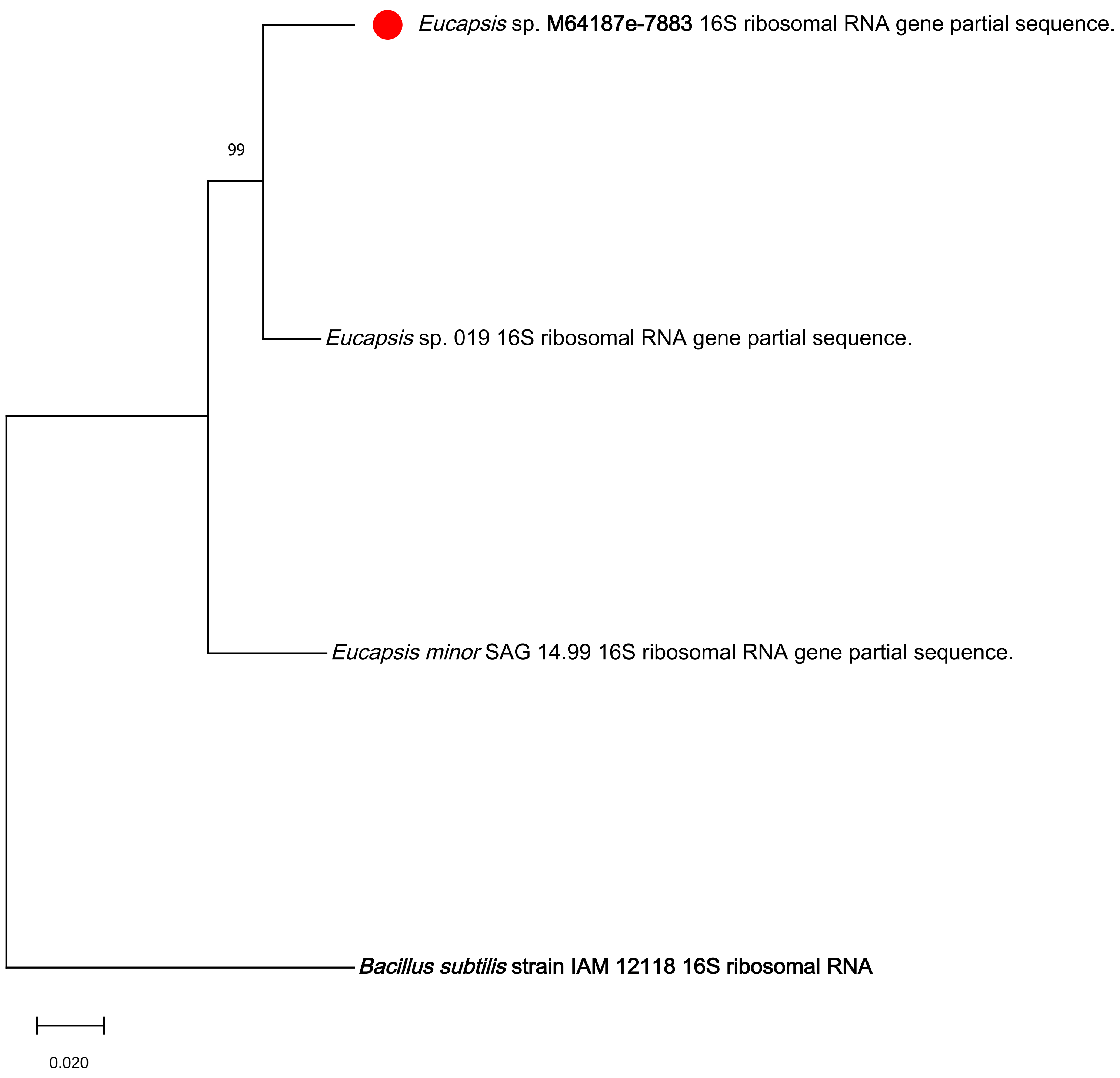

Figure S6. Topology of the 16S rRNA gene sequences of the cyanobacterial strain (*Eucapsis* sp.) isolated from the present study and its closely related sequence from the NCBI database constructed using the neighbor-joining method[46]. The percentage of replicate trees in which the associated taxa clustered together in the bootstrap test (1000 replicates) are shown next to the branches[47]. Five base substitutions for nucleotide positions are represented by the scale bar. The sequences obtained in the present study are indicated by red dot.

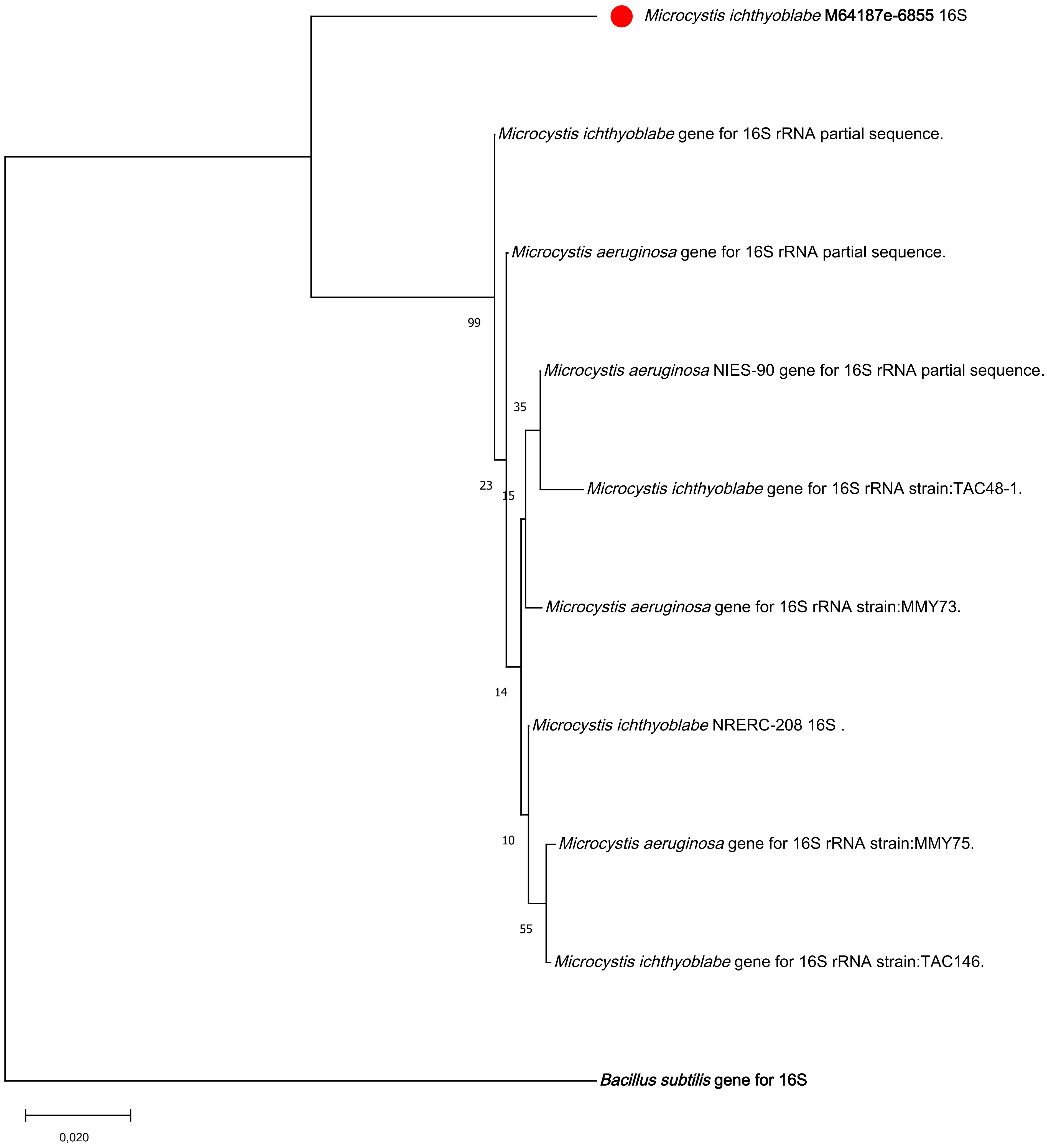

Figure S7. Topology of the 16S rRNA gene sequences of the cyanobacterial strain (*Microcystis ichthyoblabe*) isolated from the present study and its closely related sequences from the NCBI database constructed using the neighbor-joining method[46]. The percentage of replicate trees in which the associated taxa clustered together in the bootstrap test (1000 replicates) are shown next to the branches[47]. Five base substitutions for nucleotide positions are represented by the scale bar. The sequences obtained in the present study are indicated by red dot.
